# From Gene-Based Breeding to Haplotype-Based Breeding for Rice (*Oryza sativa*) Improvement in the Genomics Era

**DOI:** 10.3390/ijms27208972

**Published:** 2026-10-10

**Authors:** Trung Quoc Nguyen, Anh Quynh Ho, Ha Duc Chu, Nguyen Nguyen Chuong, Touhidur Rahman Anik, Gioi Huy Dong, Saad Sulieman, Nguyet Thi Minh Nguyen, Sudisha Jogaiah, Hong Viet La, Lam-Son Phan Tran

**Affiliations:** 1 Faculty of Biotechnology, Vietnam National University of Agriculture, Hanoi City 100000, Vietnam; nqtrung@vnua.edu.vn (T.Q.N.); hqanh.sci@gmail.com (A.Q.H.); dhgioi@vnua.edu.vn (G.H.D.); 2 Faculty of Agricultural Technology, University of Engineering and Technology, Vietnam National University Hanoi, Hanoi City 100000, Vietnam; 3 VinUni Bigdata Research Institute, VinUniversity, Hanoi City 100000, Vietnam; 4 Institute of Genomics for Crop Abiotic Stress Tolerance, Department of Plant and Soil Science, Texas Tech University, Lubbock, TX 79409, USA; chuonguy@ttu.edu (N.N.C.); tanik@ttu.edu (T.R.A.); 5 Department of Integrative Agriculture, College of Agriculture and Veterinary Medicine, United Arab Emirates University, Al Ain P.O. Box 15551, Abu Dhabi, United Arab Emirates; saadsulieman@uaeu.ac.ae; 6 Agricultural Genetics Institute, Vietnam Academy of Agricultural Sciences, Hanoi City 100000, Vietnam; nguyetha176@gmail.com; 7 Department of Environmental Science, Central University of Kerala, Kasaragod 671316, India; jsudish@cukerala.ac.in; 8 Institute of Scientific Research and Applications, Hanoi Pedagogical University 2, Phuc Yen City 35000, Vietnam; laviethong@hpu2.edu.vn

**Keywords:** rice breeding, haplotype validation, marker-assisted selection, allele pyramiding, pangenome, genomic selection

## Abstract

Rice (*Oryza sativa* L.) improvement increasingly uses genome-scale variation, but breeding decisions remain trait-oriented and depend on genetic architecture, recipient background, target environment, and selection resources. This review assesses when phased haplotype information adds value beyond causal-gene markers, single-nucleotide polymorphism assays, phenotypic selection, and genomic prediction. Reported configurations for yield, grain quality, nutrition, biotic resistance, and abiotic-stress adaptation are evaluated according to population structure, carrier frequency, effect uncertainty, haplotype-by-background interaction, haplotype-by-environment interaction, linkage drag, transferability, assay availability, and current breeding use. A three-tier hierarchy classifies configurations as Candidate, Validated, or Breeder-Ready. Most reported favorable haplotypes remain Candidate targets, fewer have controlled genetic validation, and few sequence-defined multi-variant haplotypes have documented use in active breeding programs. Practical deployment requires stable sequence nomenclature, accessible donors, diagnostic assays, elite-background and multi-environment evaluation, cost analysis, and donor-segment recovery. Graph pangenomes, phased long-read assemblies, causal reconstruction, and prospective comparisons with current selection methods can improve future assessment. Haplotype information has greatest value when phase improves a defined breeding decision and increases target-environment performance, annual genetic gain, selection efficiency, or cultivar delivery.

## 1. Introduction

Rice (*Oryza sativa* L.) supports more than half of the global population, and continued genetic improvement is required to increase production and yield stability under water limitation, salinity, nutrient-poor soils, temperature extremes, and evolving pathogens and pests [1]. Phenotypic selection and marker-assisted breeding have delivered important cultivars through genes and quantitative trait loci (QTLs) with large phenotypic effects, such as *SUBMERGENCE 1A* (*Sub1A*) for submergence tolerance [2], *PHOSPHORUS UPTAKE 1*/*PHOSPHATE-SPECIFIC TRANSPORT 1* (*Pup1/PSTOL1*) for phosphorus-deficiency tolerance [3,4], and *SHOOT K^+^ CONCENTRATION 1* (*SKC1*)-associated salinity tolerance [5]. These achievements demonstrate that trait architecture, pleiotropic effects, target environments, and product profiles should determine the breeding method. Haplotype analysis represents one molecular option within this trait-first framework and has greatest value when the phase of linked variants changes parental selection, marker design, introgression strategy, or genomic prediction. A haplotype is an ordered and phased allele sequence carried on one chromosomal homolog in a diploid individual and transmitted through a gametic chromatid unless meiotic recombination separates its components. Single-nucleotide polymorphisms (SNPs), small indels, regulatory variants, transposable-element insertions, and structural variants (SVs) may define this sequence, but these variant classes remain components of the haplotype and do not form independent transmission units. A gene haplotype comprises phased variants across a defined gene and its regulatory interval, while a block haplotype extends across a linked chromosomal segment whose boundaries are determined by sequence and recombination information. Targets located at unlinked loci or on different chromosomes form a multilocus breeding package and do not constitute one haplotype. The 3000 Rice Genomes (3K-RG) Project identified 29 million SNPs, 2.4 million small insertions and deletions, and more than 90,000 SVs across 3010 cultivated rice accessions [6,7]. Graph pangenomes and phased long-read assemblies have extended this resource through alternative sequence paths, presence-absence variations (PAVs), copy-number variation, lineage-specific genes, and non-reference sequences from cultivated and wild rice. Despite these advances, many rice haplotypes are still defined from SNPs and short indels aligned to Nipponbare, which underrepresents divergent *indica*, *aus*, aromatic, African rice, and wild *Oryza* sequences [8,9]. These resources have shifted rice genomics from isolated marker discovery toward the identification and assembly of favorable allelic combinations for defined breeding targets.

The concept of superior-haplotype breeding has gained attention after studies demonstrated that rice accessions contain favorable configurations for yield, flowering time, plant architecture, grain dimensions, amylose content, mineral accumulation, disease resistance, and tolerance to drought, salinity, flooding, heat, and cold. Analysis of 120 functionally characterized genes across the 3K-RG panel identified multiple trait-associated haplotypes and proposed their assembly into tailored rice genotypes [10]. Numerous studies have designated a haplotype as favorable or superior when its carriers show the highest phenotypic mean within a single association panel, subgroup, season, or location [10,11,12]. Such associations can be affected by population structure, local linkage disequilibrium (LD), small carrier numbers, phenotyping errors, or causal variants outside the nominated interval. Haplotype effects may also change across recipient genomes and production environments through dominance, heterosis, epistasis, pleiotropy, stress intensity, management, or pathogen populations [13,14]. Study-specific labels, such as Hap1, H4, and HapA, lack transferable sequence identity when reference assemblies, genomic coordinates, interval boundaries, or variant filters differ among studies. Sequence-based identifiers that record the reference assembly, genomic interval, phased variants, SV or PAV configuration, carrier accession, and version are needed for cross-population comparison and marker development [15,16,17,18,19]. Many rice haplotypes remain defined from SNPs and short indels aligned to Nipponbare, which limits the recovery of SVs, PAVs, copy-number differences, inversions, transposable-element insertions, and non-reference genes [7,8,9,20]. Quantitative traits, such as yield and stress recovery, frequently depend on numerous unlinked loci, and haplotype selection cannot replace genomic prediction or multi-environment field evaluation [21,22]. Packages containing several haplotypes require evaluation of dominance, epistasis, recombination probability, linkage drag, and negative pleiotropy before introgression [23,24,25]. Reported cultivar success is strongest for gene- and QTL-based deployment, while cultivar-level evidence for sequence-defined multi-haplotype packages remains limited [2,3,5,6,7]. A three-tier classification separates discovery signals from operational breeding targets. Candidate identifies a haplotype supported by a corrected association and a defined phased sequence, Validated identifies a Candidate supported by causal or strong genetic evidence and transferability tests, and Breeder-Ready identifies a Validated haplotype supported by accessible donor seed, an affordable diagnostic assay, elite-background evaluation, multi-environment performance, and documented use in an active breeding pipeline [4,24,26,27,28]. The term ‘superior’ is restricted to comparative performance within a specified product profile, genetic background, and target population of environments. Recent evidence is concentrated in Asian discovery panels, while studies of United States (U.S.) germplasm [29,30,31], International Rice Research Institute-linked populations, and international public-sector programs demonstrate the importance of regional validation, accessible markers, and program-specific cost assessment [18,21].

This review evaluates when phased haplotype information improves donor selection, marker development, introgression, pyramiding, or genomic prediction. It assesses yield and plant architecture, grain quality and nutrition, and stress adaptation, with attention to carrier frequency, effect uncertainty, haplotype-by-background interaction, haplotype-by-environment interaction, assay availability, linkage drag, and current breeding use. The final sections address evidence grading, operational deployment, sequence nomenclature, pangenome resources, and priorities for the next 5–10 years.

## 2. A Trait-First Framework for Gene-, Haplotype-, and Genome-Wide Selection in Rice

Gene- and QTL-based breeding provides the strongest cultivar-level evidence for molecular deployment in rice, as functional or tightly linked markers have supported *SEMI-DWARF1* (*sd-1*) for lodging resistance [32], *WAXY* (*Wx*, which encodes granule-bound starch synthase I) and *ALKALI DEGENERATION/STARCH SYNTHASE II-A* (*ALK/SSIIa*) for cooking quality, Betaine aldehyde dehydrogenase 2 (*BADH2*) for fragrance [33], *Sub1A* for submergence tolerance [2,26], and combinations of *xa5*, *xa13*, and *Xa21* for bacterial blight resistance [31,34,35,36]. Marker-assisted backcrossing produced Improved Samba Mahsuri [35], Improved Pusa Basmati 1 [37], Swarna-Sub1 [26], and DRR Dhan 58 [38], among other released materials. These cases should be distinguished from haplotype-based deployment unless phased multi-variant configurations served as the actual selection units. Haplotype analysis is most useful when linked variants define functional alleles that one diagnostic site cannot resolve, as illustrated by *Wx* [39] and *Pup1/PSTOL1* [3,40]. For traits controlled by numerous unlinked loci, validated haplotypes may enter genomic prediction as fixed or weighted effects only after comparative analyses demonstrate improved prediction accuracy or genetic gain [21,22,41].

A haplotype is an ordered, phased allele combination carried on one chromosomal homolog across a defined interval, while the paired haplotypes of a diploid individual form its diplotype. SNPs, indels, transposable-element insertions, copy-number changes, SVs, and PAVs may define gene, block, or structural haplotypes, but targets at independently assorting loci form a multilocus breeding package. Block boundaries depend on population linkage disequilibrium and recombination history, so stable reporting requires sequence-defined variants and genomic coordinates [7,8,9,11,25]. “Favorable” denotes movement toward a specified breeding goal, while “superior” denotes performance above defined comparators within specified genetic backgrounds, environments, and product requirements [10,39,42]. A multi-site haplotype assay is justified only when it improves donor discrimination, selection accuracy, genetic gain, or cost relative to a causal marker or SNP model.

The concepts give haplotype-based breeding greater allelic resolution than gene-centered selection, since phased combinations of promoter variants, coding polymorphisms, insertions, deletions, and SVs can distinguish functional forms that would otherwise receive the same gene label. The principal differences between these breeding frameworks and the transition from single-gene identification to superior-haplotype deployment are summarized in Figure 1. Haplotype analysis first improves allele classification by separating accessions that possess the same target gene but differ in transcriptional regulation, protein sequence, linked functional sites, or phenotypic effect. Analysis of 120 functionally characterized rice genes across the 3K-RG panel showed that more than 75% contained two to fifteen haplotypes, and field evaluation identified superior haplotypes for 21 genes associated with flowering time, plant height, tiller number, panicle architecture, grain yield, grain shape, amylose content, and grain mineral concentration [10]. This resolution was evident at *Ghd7*, where 14 haplotypes separated early-, medium-, and late-flowering accessions, and at *GS5*, where distinct haplotypes were associated with slender, medium-slender, or bold grains. Haplotype-frequency analysis also identifies favorable diversity that is poorly represented in elite breeding materials, as *OsNAS3*-H2 was associated with greater grain iron and zinc concentrations but occurred in only 1% of the surveyed accessions, which identifies a restricted donor group that could be overlooked through selection based on gene presence. A second advantage concerns parental selection, since breeders can compare the complete haplotype profiles of elite lines and donors, identify favorable haplotypes missing from a target breeding population, and choose parents with complementary allelic combinations for yield, maturity, grain quality, and stress adaptation. A donor that carries several desired haplotypes can reduce the number of crosses required, whereas haplotype maps can reveal unfavorable variants linked to a target allele before introgression. Multi-site haplotype markers also permit recombinant selection at both sides of the target interval, which helps preserve the favorable variant combination and restrict the transfer of unwanted donor segments associated with linkage drag. The value of this approach becomes greater for quantitative and environmentally responsive traits, where phenotypic performance may depend on several variants within a gene and favorable combinations across multiple loci.

## 3. Evidence-Ranked Genetic Configurations for Major Rice Breeding Traits

### 3.1. Superior Haplotypes for Yield, Plant Architecture, and Flowering Adaptation

Grain yield arises from a coordinated relationship among panicle number, grain number per panicle, grain weight, tiller productivity, plant height, panicle form, and crop duration, and improvement of one component may reduce another through competition for assimilates or changes in plant development. Table 1 summarizes reported favorable haplotypes across these component traits, although only a limited number have been evaluated in defined combinations. Bulked-segregant sequencing of an F_5_ population identified three major QTL intervals for effective panicle number, while expression and 3K-RG haplotype analyses narrowed the candidates to five genes [43]. GWAS under different nitrogen gradients further identified five candidate genes whose haplotypes differed significantly in effective tiller number, which indicates that tillering haplotypes should be evaluated under the nutrient regime of the target production system [44]. In the 3K-RG panel, *SD1-H8* occurred in 30.13% of the accessions and was associated with semi-dwarf stature, while *MOC1-H9* and *IPA1-H14* occurred at frequencies of 23.08% and 6.64% and were associated with productive tiller number [10]. The same analysis associated *DEP1-H2* with panicle length, *LAX1-H5*, *LP-H13*, and *OSH1-H4* with primary panicle branching, and *PHD1-H14*, *AGO7-H15*, and *ROC5-H2* with greater single-plant yield across two field seasons [10]. These panel-defined classes belong to the Candidate tier, as two-season association does not establish causal identity, transferability across breeding populations, or suitability for operational selection. Panel-defined *IPA1-H14* must also remain distinct from the semidominant functional alleles of *OsSPL14/IPA1* that disrupt miR156-mediated regulation [45], while *SD1-H8* should not be treated as identical to the causal Green Revolution allele of *sd1* [46].

Controlled genetic studies provide stronger evidence for loci that regulate complementary yield components. Reduced expression or loss of function at *Gn1a/OsCKX2* increases cytokinin accumulation in inflorescence meristems and promotes reproductive-organ formation [56], while favorable variation at *DEP1* produces compact erect panicles with shorter internodes and greater branch and grain numbers [57]. *IPA1/OsSPL14* modifies tiller number, panicle branching, culm strength, and lodging resistance, which makes its value dependent on the architectural requirements of the recipient cultivar [45]. At the whole-plant level, the *EPN4-1* haplotype of *EPN4/NAL1* coordinated effective panicle number, grain number, and grain weight, while its combination with *GNP1-6* increased grain yield [52]. Additional functional evidence has been reported for *MOG1*, whose natural variation regulates grain number and grain weight [63], and for *GNP3/OsMKKK22*, whose favorable *Hap-T* increased grain number and panicle branching in NIL and functional analyses [64]. Complementation and functional assays supported favorable variation at *GNP2/OsGSK1* and *GNP5*, while the Type I *GNP2-GNP5* combination produced the highest grain number and yield in the evaluated *japonica* materials [65]. NIL and field analyses also supported *Hap-STRONG1^C^* as a Validated configuration for stronger culms, improved architecture, lodging resistance, and yield [66], while GWAS and CRISPR analysis supported the role of *EPE1/OsGA2ox9* in panicle elongation [53]. These cases have stronger evidence than panel associations and may enter the Validated tier within the tested genetic materials, but their value remains conditional on maturity, plant height, recipient background, and production environment.

Grain size and weight must be evaluated with panicle sink capacity, lodging resistance, and market requirements. Natural variation in *OsSWEET11* and *OsSWEET15* among 139 Yunnan landraces was associated with differences in grain traits and provides Candidate haplotypes related to sucrose transport during grain filling [54]. Functional variation at *GS3* modifies grain length, while *GW2* and *GW5/qSW5* [61] regulate grain width or weight through changes in cell proliferation and grain development [60,61,62]. Candidate-gene analysis associated *AET1-H2*, *SNAC1-H1*, *qTGW3-H1*, *DHD1-H2*, and *GW5-H1* with thousand-grain weight, with reported PVE values of 15.01%, 14.78%, 13.48%, 10.78%, and 5.88%, respectively [48]. The TGW-HC4 configuration combined *SNAC1-H1*, *qTGW3-H1*, *AET1-H2*, and *DHD1-H2* and occurred in 12 of 180 accessions, which represented 6.67% of the evaluated panel and was associated with a mean thousand-grain weight of 29.47 g [48]. These associations define useful parental-screening targets, but TGW-HC4 should remain in the Candidate tier until its combined effect is reproduced through controlled segregation, independent genetic backgrounds, and multi-environment trials. Experimental pyramiding of *gn1a*, *gs3*, and *ipa1* in Zhonghua 11 showed that *gn1a-gs3* and *gs3-ipa1* increased grain yield relative to the corresponding single mutants, while *gs3-ipa1* and the *gn1a-gs3-ipa1* combination increased grain weight [23]. The pyramided plants also showed increased height, which demonstrates that gains in grain number and weight may introduce lodging risk or alter plant type. Recombinant-line studies provide further evidence that favorable effects can change after allele combination [24]. Evaluation of a yield package must include grain shape, grain weight, maturity, plant height, lodging resistance, linkage drag, recombination probability, population-size requirements, and negative pleiotropy.

The distribution and performance of yield-related haplotypes differ among rice genetic groups. The favorable *Gn1a* allele was identified in the *indica* cultivar Habataki, *IPA1* alleles occur in *indica* and *japonica* materials, and truncated *DEP1* alleles have been used extensively in erect-panicle *japonica* cultivars. Population analyses identified subgroup-specific LD and adaptation patterns at flowering-time and architectural loci [11,13,47], which can cause one haplotype label to represent different linked sequences across *indica*, *japonica*, *aus*, aromatic, or admixed populations. At *GS3*, nine haplotypes were identified among 160 wild accessions and representative cultivars, and the ancestral *GS3-H4* occurred in 88 wild accessions as well as four *aus* and four aromatic cultivars [55]. This distribution identifies wild and traditional germplasm as a source of uncommon alleles, but rarity and ancestral origin do not demonstrate a favorable effect after introgression into elite material. Regional evaluation of *GS3* and *qGL7.1* haplotypes in 323 elite U.S. rice genotypes further showed that haplotypes derived from international panels require validation within regional grain classes and breeding populations [30]. These differences represent H×B effects that may arise from modifiers, epistasis, local LD, donor-segment composition, or differences in the frequency of interacting alleles. A rare or geographically restricted haplotype should remain a Candidate donor until its effect is reproduced in contrasting elite recipient genomes.

Heading-date loci provide a direct example of H×E interaction and demonstrate why a yield-related allele cannot be separated from adaptation. Strong functional alleles of *Ghd7* delay heading under long-day conditions and increase plant height and panicle size, while *Ghd8/DTH8* influences heading date, plant height, and grain productivity [23,47]. Within the 3K-RG analysis, *Ghd7-H8* occurred at a frequency of 3.08% and was classified as early flowering, *Ghd7-H14* occurred at 42.60% and was associated with intermediate flowering, and *Ghd7-H6* occurred at 1.65% and was associated with later flowering [10]. These categories apply to the populations and field conditions used in the original analysis and cannot be transferred directly across latitudes, planting dates, or cropping systems. Near-isogenic populations carrying combinations of *Ghd7*, *Ghd8*, *OsPRR37*, and *Hd1* showed more than 95 days of heading-date variation under natural long days and 42 days under natural short days [13]. The strongest two-locus interaction changed from *Ghd7 × Ghd8* under long days to *Ghd7 × OsPRR37* under short days, which provides direct evidence that photoperiod modifies epistatic relationships among heading-date loci [13]. Nitrogen supply can create an additional environmental dependency through its effects on tiller production, plant height, lodging, and crop duration [44]. The preferred haplotype configuration depends on latitude, planting season, temperature regime, crop density, nutrient supply, water availability, and the maturity requirement of the target product profile. Multilocation and multiseason evaluation across relevant elite backgrounds is required before a heading-date or yield haplotype can advance from Candidate to Validated or Breeder-Ready.

Table 1 shows that most yield-related configurations remain Candidate targets and that Breeder-Ready multi-haplotype packages are scarce. Validated large-effect loci can be fixed with diagnostic assays, while genome-wide selection and field evaluation can improve the remaining polygenic background. A practical yield package must balance effective panicle number, grains per panicle, grain weight, culm strength, canopy architecture, flowering time, and regional market requirements without unacceptable penalties in maturity, height, lodging, or grain quality. Large-effect Validated loci may be fixed through diagnostic markers, while the remaining polygenic background should be improved through genome-wide selection and field testing as discussed in Section 4.4. The breeding value of each package should be judged through selection accuracy, recovery probability, assay cost, genetic gain per cycle, multi-environment yield, and documented advancement within an active breeding program.

### 3.2. Superior Haplotypes for Grain Quality and Nutritional Traits

Grain quality comprises appearance, milling performance, cooking properties, texture, aroma, and nutritional composition, although no genetic configuration can improve every component under all production and market conditions (Table 2). Selection should begin with a defined product profile that identifies the preferred grain dimensions, amylose range, gelatinization temperature, cooked-rice texture, aroma class, milling standard, nutritional target, and acceptable yield level. A quality-associated haplotype detected through GWAS or haplotype-phenotype analysis belongs to the Candidate tier until independent genetic support becomes available, while NILs, edited lines, transgenic complementation, reciprocal introgression, or validated recombinant materials can support placement in the Validated tier. Breeder-Ready classification requires an accessible donor, a diagnostic assay, elite-background evaluation, multi-environment performance, processing tests, sensory assessment, and documented use within an active breeding program. This product-based framework is essential for grain quality, since a high-amylose allele may benefit dry-cooked rice or processed foods but reduce acceptance in markets that prefer soft, cohesive rice, while lower grain protein may improve eating quality but conflict with nutritional enrichment.

Appearance quality clearly illustrates the distinction between association evidence and controlled validation. The *Chalk9*-L haplotype contains a causal 64-bp promoter insertion that increases *Chalk9* expression and promotes degradation of OsEBP89, which reduces abnormal accumulation of storage compounds within chalky endosperm regions [75]. Its genetic effect was examined through an *indica* GWAS panel, a NIL that carried *Chalk9*-H from Kasalath in the Nipponbare background, transgenic tests in Guichao2, and promoter editing in Nipponbare. Introduction of the *Chalk9*-L promoter reduced chalky grain rate by approximately 20% and degree of chalkiness by approximately 40% without a detected yield penalty in the tested materials. *Chalk9*-L occurred in all 123 surveyed *japonica* cultivars and in 81% of 105 elite *indica* cultivars released in China during the preceding decade, while its frequencies differed among *indica* subgroups, *aus*, and aromatic rice [75]. These results support Validated classification, although performance under high temperatures during grain filling and implementation across additional elite backgrounds are required before Breeder-Ready designation [75]. The GBSSI/*Wx*-*SSIIa/ALK* configuration GGAAC-GGC was associated with low chalkiness and high head-rice recovery under heat in a 60-accession panel across two seasons, but the absence of controlled introgression or causal reconstruction retains this configuration in the Candidate tier [42]. A rare *Chalk7-GS3-Chalk5* combination improved grain appearance in derived breeding materials, while its limited donor frequency and possible effects on grain dimensions require market-specific evaluation [59].

Amylose content, gelatinization temperature, and gel consistency are major determinants of eating and cooking quality, with *Wx* and *SSIIa/ALK* serving as central targets for product-specific selection. Variation at *Wx* changes amylose synthesis and cooked-rice texture, while interaction between *Wx* and *SSIII-2* modifies physicochemical properties across genetic materials [67]. The *Wx* configurations TT-AA-CC and TT-AA-TT produced amylose contents of 6.09% and 8.03%, with gel-consistency values of 114.08 and 111.87 mm, respectively, in a segregating population. These configurations suit soft-rice products but remain Candidate records until their phased variants, background effects, and multi-location performance have been independently confirmed. *RSR1*-H8 was associated with intermediate amylose content and occurred in 4.20% of the 3K panel, while Candidate haplotypes at *Os06g0232700*, *Os06g18710*, *Os06g18720*, *Os06g0229000*, *Os06g12380*, and *Os10g046900* were associated with amylose content, alkali-spreading value, or gel consistency in a 150-accession subset [10,68]. The frequencies and effects of these haplotypes may differ among *indica*, *japonica*, *aus*, and aromatic groups due to population structure and subgroup-specific LD, which limits direct transfer of a study-defined H1, H4, or H14 label to another breeding population.

Resistant starch and predicted glycemic index extend quality selection from sensory properties to nutritional function. *Os06g11100-H4* and *Os06g16080*-H11 were associated with resistant starch contents of 3.28% and 5.37%, while* Os02g16970*-H14 was associated with a resistant starch content of 4.49% and *Os08g12590*-H13 was associated with a predicted glycemic index of 62.52 [68]. These records remain in the Candidate tier, since their effects were derived from association and physicochemical analyses in a limited 3K subset without causal reconstruction or independent elite-background testing. The *Wx*^a^-*SSIIa*^G-TT^ configuration identified in CG133R × Javanica 22 recombinant inbred lines increased cooked and retrograded resistant starch while maintaining comparatively acceptable cooking properties [69]. This multilocus configuration represents an advanced Candidate package because QTL and recombinant-line evidence supports its breeding potential, although confirmation across different starch backgrounds, production environments, processing methods, and sensory panels remains necessary. Selection for resistant starch should include amylose content, gelatinization, retrogradation, grain hardness, cooking time, and consumer acceptance, since improvement in one nutritional measurement can alter several processing and sensory traits.

Aroma and grain protein illustrate two contrasting routes from functional validation to product development. The fragrance-associated *BADH2/fgr*-Hap6 configuration promotes accumulation of 2-acetyl-1-pyrroline and occurred in 5.8% of the full 3K panel but in approximately 51% of the aromatic subgroup. Global haplotype analysis, aroma measurements, and a Kompetitive Allele-Specific PCR (KASP) assay tested in U.S. rice support classification as Validated and marker-ready, although the configuration should be deployed within aromatic product profiles that retain the required grain shape, cooking quality, and agronomic adaptation [29]. *OsGATA7*-Hap1 and *SMOS1*-Hap1 reduce expression of *OsGluA2*, lower grain protein accumulation, and improve taste scores, with support from association analysis, CRISPR materials, and introgression lines [76]. These configurations qualify as Validated for premium table rice, while *OsAAP6/qPC1* and *OsGluA2/qGPC-10* provide validated targets for higher protein and amino acid contents through NIL, transgenic, QTL-cloning, and gene-editing evidence [65,66]. The opposing effects of these loci prevent a universal ranking, since lower protein may favor soft texture and taste while higher protein may favor nutritional products, institutional diets, or biofortified rice.

Mineral and amino acid enrichment requires simultaneous assessment of nutritional gain, environmental stability, and undesirable element accumulation. *OsNAS3*-H2 was associated with increased iron and zinc but occurred in only 1% of the 3K panel, which identifies a rare Candidate donor that requires controlled introgression and background recovery [10]. Os06g0489500-Hap3 increased iron and zinc without a detected increase in cadmium in an *indica* panel, while Os07g0568300-Hap4 and Hap8 increased zinc and Hap6 increased iron in *indica* or *japonica* materials [70]. The subgroup-specific effects at Os07g0568300 provide evidence of H×B and support separate validation within target *indica* and *japonica* breeding pools. Deepwater rice from Assam contained an ACCC haplotype associated with higher grain zinc and a CT configuration associated with grain oil content, which expands donor discovery beyond frequently studied Asian elite panels [71]. Multi-model GWAS identified loci associated with lysine accumulation and detected QTN-by-environment effects, which demonstrate that nutritional ranking can change across production environments [72]. Pigmented landraces provide additional Candidate resources, including *Rc*-H002 for phenolic content and antioxidant capacity in Shanlan upland rice [73], as well as *OsKala4*-H01 and *OsMYB3*-H01 for black pericarp and seed anthocyanin accumulation [74]. Evaluation of these materials should include yield, maturity, mineral bioavailability, cadmium concentration, pigment stability after cooking, and consumer acceptance. Table 2 identifies a limited set of Validated targets, including *Chalk9*-L, *BADH2/fgr*-Hap6, *OsGATA7*-Hap1, *SMOS1*-Hap1, *OsAAP6*, and *OsGluA2*, while most nutrition-related configurations remain Candidate resources. Product-specific packages should proceed only after the component loci have compatible effects on yield, milling performance, cooking quality, sensory properties, and adaptation.

### 3.3. Superior Haplotypes for Biotic Resistance and Abiotic Stress Tolerance

Stress-adaptive haplotypes differ from yield- and quality-related haplotypes in the conditions required for their evaluation. Their phenotypic value depends on stress timing, intensity, duration, developmental stage, and recovery after exposure. Disease and insect resistance also vary with pathogen isolate or pest biotype. The most useful haplotypes are therefore those supported by functional validation and field performance, particularly when they protect yield without imposing a marked penalty under favorable conditions. Association-derived haplotypes belong to the Candidate tier, controlled genetic and functional evidence can support the Validated tier, and Breeder-Ready classification requires diagnostic assays, accessible donors, elite-background evaluation, multi-environment performance, manageable linkage drag, and documented integration into an active breeding pipeline (Table 3).

Drought-related configurations show a wide range of evidence quality. *DROT1*-Hap3 contains a promoter C-to-T variant at position s18975900 that increases *DROT1* expression and supports survival, biomass maintenance, and yield-related performance under field water deficit. The haplotype was identified in a 743-accession panel and was enriched in upland tropical *japonica* and upland *indica* germplasm, while GWAS, introgression lines, CRISPR materials, expression analyses, and field trials support its classification as Validated [87]. *DRG9*-Hap1 occurred in 130 of 503 accessions and improved relative green-projected area, survival, and drought resistance after transfer into Huanghuazhan. Functional analyses connected DRG9 activity with stress-responsive protein condensates, which provide mechanistic and introgression evidence for Validated classification [88]. In comparison, *OsGSK1*-H4, *OsDSR2*-H3, *OsDIL1*-H22, *OsDREB1C*-H3, *ASR3*-H88, *DSM3*-H4, and *ZFP182*-H4 were associated with higher single-plant yield under reproductive-stage drought in a 399-accession *indica* and *aus* panel across two seasons. Reported yield advantages among these haplotypes ranged from 8.5% to 62.5%, although low carrier numbers, population structure, and the absence of NIL or edited-line confirmation retain these records in the Candidate tier [79]. *ASR3*-H88 occurred in only three accessions, which makes effect estimation uncertain and identifies the configuration as a rare donor-screening target.

Salinity tolerance requires separate assessment of osmotic stress, sodium toxicity, developmental stage, and soil heterogeneity. *OsSTL1*-Hap1 and *OsSTL2*-Hap1 or Hap2 were associated with seedling salt tolerance in a 664-accession panel, while the combined configuration Co-hap2, composed of *OsSTL1*-Hap1 and *OsSTL2*-Hap2, occurred in 10 of the 11 accessions with the highest tolerance scores [80]. The favorable lead allele at *OsSTL1* occurred in 98.5% of *indica* but only 23.0% of *japonica*, while the corresponding *OsSTL2* allele occurred in 99.3% of *indica* and 78.0% of *japonica*, which demonstrates major H×B differences and unequal opportunities for selection within each subgroup. These configurations remain Candidate targets until their individual and non-additive effects are tested in segregating materials under reproductive-stage and field salinity. *OsWRKY53* haplotype A supported higher plant water content than haplotype B under salt exposure, and GWAS, CRISPR, and ion-homeostasis assays support Validated classification within the tested materials [89]. Sequence analysis of *BADH1* across 475 accessions identified 39 haplotypes, with Hap_2, Hap_18, and Hap_23 associated with salt-adapted *indica* germplasm [81], while analysis of 233 Thai accessions identified 79 *OsCUL3c* haplotypes and a specific configuration associated with salt responses [82]. Both records remain Candidate targets due to limited controlled genetic evidence. Nutrient-related constraints require similar caution, since Hap1 at a phosphorus-use-efficiency candidate gene performed better under acidic, low-phosphorus soil [83], while gene-based haplotypes associated with iron-toxicity tolerance were identified through integrated GWAS [84]. Soil chemistry, mineral availability, microbial activity, and water regime can alter these effects across locations.

Flood adaptation comprises at least two genetically distinct phases, which are anaerobic establishment and survival during complete submergence. The FR13A-derived *Sub1A-1* allele restricts excessive shoot elongation, conserves carbohydrate reserves, promotes survival during prolonged inundation, and supports rapid recovery after floodwater withdrawal [2]. Backcross selection with markers transferred *Sub1A-1* into elite recipient cultivars and supported field deployment in flood-prone production systems [26], while sequence analysis across diverse cultivars documented allelic variation at *Sub1A*, *Sub1B*, and *Sub1C* [96]. *Sub1A-1* qualifies as a Breeder-Ready allele benchmark, although this deployment record does not assign equivalent status to study-defined multi-variant haplotypes. *OsTPP7*-Hap_1 addresses anaerobic germination through longer coleoptiles and a higher flooding-tolerance index during early establishment. It occurred in 395 of 475 accessions and was concentrated in *japonica* germplasm, but its evidence remains Candidate due to the absence of controlled introgression and field validation across direct-seeded systems [85]. A product designed for flood-prone direct seeding may require both establishment and submergence components, while recombination, maturity, seedling vigor, and yield must be examined within the intended recipient background.

Temperature adaptation also displays strong subgroup differentiation and developmental-stage specificity. *SLG1*-Hap2, also described as *SLG1*-Ind, occurred in 93.9% of 2691 *indica* accessions and conferred thermotolerance at seedling and reproductive stages. Reciprocal NILs showed survival rates of approximately 81% and 48% for the tolerant and sensitive alleles, while complementation and transgenic analyses support Validated classification [90]. *TTL1*-HapL was identified through a rice super-pangenome and was associated with seedling thermotolerance, longer grains, and heavier grains in a 187-accession core panel. Functional evidence supports the locus, while the pangenome-based discovery reduces reference bias that can conceal structural or presence-absence variation [91]. Cold tolerance at the booting stage depends on a different genetic profile. *CTB3*-Hap1 increased seed-setting rate under cold exposure in a 525-accession panel, and promoter-indel analysis, complementation, CRISPR, and NIL tests support Validated classification. The combination of *OsTCP19*-T-Hap1 and *CTB3*-Hap1 produced the highest seed-setting rate but was detected only in *japonica* accessions, which identifies a useful cold-tolerance package and a major H×B limitation for transfer into *indica* [92]. Backcross design for this package should control heading date, reproductive compatibility, donor-segment length, and *japonica*-linked effects.

Biotic resistance requires evidence across representative pathogen populations and pest biotypes, since resistance conferred by one receptor may fail after a shift in the attacking population. The linked PigmR-PigmS NLR configuration provides a model of non-additive control, since PigmR recognizes multiple blast isolates while PigmS moderates the reproductive cost associated with strong immune activation through epigenetic regulation [93]. Map-based cloning, NILs, transgenic materials, and field evaluation support Validated classification, although regional isolate testing and diagnostic marker development remain necessary. At the *BPH1/9* NLR locus, 21 allelotypes were identified among 117 varieties and landraces, while *BPH1/9-7* from T12 and *BPH1/9-9* from Pokkali conferred resistance to brown-planthopper biotypes 1, 2, and 3 [83]. *STV11*-R and *STV11*-R′ provide durable resistance to rice stripe virus and occurred in 85.03% of surveyed *indica* cultivars, while their lower frequency in *japonica* identifies a defined introgression opportunity [95]. In contrast, LOC_Os12g39700-H4 and LOC_Os06g30440-H33 for blast resistance, along with LOC_Os02g12660-H39 and LOC_Os05g35490-H49 for bacterial leaf blight resistance, were identified through GWAS and haplotype-phenotype analysis in a 147-accession subset and remain Candidate donor-screening targets [86]. Resistance spectra, isolate specificity, lesion development, yield cost, and linkage with undesirable donor segments require controlled evaluation before deployment. Marker-assisted transfer of *xa5*, *xa13*, and *Xa21* into PR106 and Samba Mahsuri provides an operational benchmark for disease-resistance gene packages, although these unlinked loci should not be described as one haplotype [34,35]. Stress-resistance packages should be designed for defined production systems and should combine only targets that retain yield, maturity, grain quality, and resistance durability across representative trials.

## 4. Evidence Grading and Operational Deployment in Rice Breeding

### 4.1. Three-Tier Evidence Hierarchy

The practical value of haplotype research depends on a classification that separates statistical discovery, genetic validation, and routine breeding use, since the terms favorable, superior, functionally validated, and breeder-validated describe different levels of evidence and should not be treated as equivalent [97]. We adopt three evidence tiers that apply to sequence-defined haplotypes, functional alleles, structural variants, linked genomic intervals, and multilocus configurations. Candidate identifies a configuration supported mainly by association analysis, haplotype-phenotype comparison, or population distribution, while Validated identifies a configuration supported by independent confirmation and causal or strong genetic evidence from near-isogenic lines, recombinant progenies, reciprocal introgression, complementation, or genome editing. Breeder-Ready identifies a Validated configuration that also has accessible donor material, a diagnostic assay, a defined selection protocol, acceptable agronomic performance in elite material, manageable linkage drag, and documented use within an active breeding program.

The three tiers form an evidence pathway in which Candidate configurations support donor screening and causal testing, Validated configurations support controlled introgression and pre-breeding, and Breeder-Ready configurations support routine selection within a defined breeding program (Figure 2). Advancement from Candidate to Validated requires confirmation of the genetic effect outside the discovery panel, identification of the causal variant or a tightly linked diagnostic variant, and evaluation of transferability across recipient backgrounds and target environments. Advancement to Breeder-Ready requires operational evidence that covers donor-seed availability, assay accuracy, genotyping cost, population-size requirements, selection records, agronomic penalties, and prospective genetic gain. Tier assignment can move downward when later experiments identify background dependence, environment sensitivity, undesirable pleiotropy, linkage drag, recombination failure, or poor assay performance, which means that Breeder-Ready status applies to a specified breeding program and target population of environments.

The evidence hierarchy changes how published examples should be used. Particularly, *OsGSK1*-H4 remains a Candidate configuration since its drought association was identified within a diversity panel without haplotype-specific causal confirmation [79], while *DROT1*-Hap3 reaches the Validated tier through association analysis, promoter-variant identification, introgression, genome editing, expression analysis, and drought evaluation [87]. The 64-bp promoter indel defining *Chalk9*-L also meets the Validated criteria through near-isogenic, transgenic, and introgression evidence [75]. The *BADH2/fgr* fragrance haplotype and validated markers for *GS3* and *qGL7.1* demonstrate marker-ready configurations, although assay availability does not confer Breeder-Ready status without program-level deployment records [29,30]. The functional *Sub1A-1* allele provides a Breeder-Ready benchmark through marker-assisted backcrossing, evaluation in several elite recipient backgrounds, and successful cultivar development in flood-prone production areas [2,26].

### 4.2. Population Structure, Linkage Disequilibrium, Genetic Background, and Environment

Candidate discovery should apply statistical models that account for kinship and population structure, while each analysis should report haplotype carrier number, subgroup frequency, effect size, uncertainty, multiple-testing correction, phenotyping conditions, and the reference haplotype used for comparison [97]. Subgroup-specific analyses are essential when favorable classes are concentrated within *indica*, *japonica*, *aus*, aromatic, or admixed germplasm, since a significant pooled-panel effect may originate from population differentiation instead of a causal haplotype. Rare haplotypes should remain eligible for validation when their effects are biologically important, although their estimates require confidence intervals, independent carriers, and targeted donor evaluation.

LD structure varies among rice subpopulations and determines whether a reported haplotype represents a causal configuration or a population-specific marker for a nearby locus. Local ancestry analysis, conditional association tests, recombinant evaluation, and phased sequence comparison can distinguish the target configuration from linked donor segments. Transfer to another breeding population requires reconstruction of the phased variant string within the stated genomic interval, since labels such as Hap1, H4, or HapA have no stable meaning outside the study in which they were assigned. The reference assembly, genomic coordinates, variant order, phase, structural-variant class, carrier accession, and haplotype version should accompany every reported label.

H×B describes variation in haplotype effects across recipient genomes due to modifiers, dominance, heterosis, epistasis, or linked donor segments, while H×E describes variation across locations, seasons, management systems, stress intensities, developmental stages, and pathogen or pest populations. A minimum transferability design should contain at least two contrasting elite backgrounds, relevant non-stress controls, and multi-location or managed-stress trials that measure yield, maturity, grain quality, recovery, reproductive performance, and correlated penalties. Hybrid programs also require diplotype evaluation within the intended male and female pools, since additive effects estimated from inbred accessions may not predict heterozygous performance.

### 4.3. Operationalizing Haplotype Deployment Through Marker Conversion, Cost Analysis, and Background Selection

Marker conversion should follow the biological structure of the target configuration. One or several causal SNPs or short insertions and deletions can often be converted into KASP assays, while longer phased segments may require multiplex amplicon genotyping, targeted sequencing, or several diagnostic markers. Structural variation, presence-absence variation, gene copy number, and resistance-gene clusters may require junction-specific, dosage-sensitive, or sequence-based assays. Each assay should be evaluated for parental polymorphism, agreement with sequence data, call rate, missing-data frequency, segregation ratio, error rate, plate-to-plate performance, and compatibility with the breeding program’s germplasm. The 1K-RiCA amplicon panel [18], rice 6K SNP array [98], trait-specific KASP resources [99], and high-density public-sector KASP panels [100] illustrate complementary options for rice breeding.

Genotyping cost should be calculated per correct selection decision and should cover assay development, DNA extraction, consumables, plate utilization, failed reactions, repeated assays, data management, staff time, and changes in field-testing expenditure. KASP is suitable for a small number of targets evaluated across large populations, while multiplex amplicons, SNP arrays, or low-coverage sequencing become more economical when many loci and genome-wide background information are required. Platform selection should consider the number of targets, annual sample volume, laboratory access, procurement time, technical expertise, data-processing capacity, and the economic value of the phenotyping stage that marker selection can replace.

Operational introgression should combine foreground selection for the target configuration, recombinant selection around the donor interval, and background selection for recovery of the elite recipient genome. Required family size must be estimated from target number, linkage relationships, recombination frequency, assay error, and the probability of recovering the complete package, since excessive locus numbers can make the crossing design impractical. A national program can support this process through a shared marker service, region-specific target product profiles, version-controlled donor and assay catalogues, harmonized data records, and stage gates that restrict large-scale crossing to configurations that have passed the required evidence tier. Candidate configurations should remain within donor screening and causal evaluation, while Validated configurations can enter controlled pre-breeding and Breeder-Ready configurations can enter routine selection.

### 4.4. Haplotype Pyramiding and Integration with Genomic Selection

Haplotype pyramiding should begin with a product profile that defines production system, market class, maturity, grain quality, stress exposure, and agronomic performance. Cross design should account for epistasis, linkage drag, recombination suppression, pleiotropy, and package-recovery probability. Complete packages and informative subsets require comparative tests before low-value or antagonistic components can be excluded, as illustrated by the balance between resistance and reproductive cost in the linked PigmR-PigmS pair [93]. For polygenic traits, SNP-based genomic selection provides the comparison baseline [21]. Haplotype-aware models may capture local linkage disequilibrium, multi-allelic variation, or local epistasis, but rice studies report trait- and population-dependent gains [41]. Prospective comparisons should use identical training sets, validation folds, environments, genotypes, and candidates and should report prediction accuracy, computing demand, genotyping cost, cycle length, response, and annual genetic gain.

### 4.5. Pangenomes, Haplotype Nomenclature, and Interoperable Data

Variant discovery against a single Nipponbare reference underrepresents sequences that are divergent or absent in *indica*, *aus*, aromatic, African rice, and wild *Oryza* accessions, particularly structural variation, presence-absence variation, gene copy-number differences, inversions, and complex resistance-gene clusters [7,8,9,20,25]. Graph pangenomes and phased long-read assemblies represent alternative sequence paths and connect SNPs, insertions and deletions, regulatory regions, and structural variants across complete homologous segments. These resources improve the definition of pan-haplotypes and reduce reference bias, although phenotype quality, causal testing, transferability assessment, and conversion into affordable assays remain necessary before breeding use [8].

A stable nomenclature should assign each rice haplotype a concise registry identifier linked to a machine-readable record that specifies the reference assembly, chromosome, genomic start and end coordinates, phased variant sequence, structural-variation or presence-absence-variation class, representative carrier accession, and record version. This approach provides a compact identifier for scientific reporting while preserving the sequence definition and provenance information required for comparison across studies and breeding populations. The registry should store normalized alleles, coordinate conventions, phase, sequence checksum, alternative-reference mappings, provenance, subgroup frequency, effect estimate, uncertainty, H×B and H×E results, donor-seed source, diagnostic assay, linkage information, agronomic penalties, evidence tier, and current breeding use. Study-specific labels such as Hap1 or HapA may remain as aliases, although they should not replace the sequence definition. Rice SNP-Seek supports genotype, accession, gene, and marker queries across the 3K Rice Genomes panel [15], whereas Rice Functional and Genomic Breeding database v2.0 connects germplasm, phenotype, haplotype, SNP, indel, and reconstructed sequence information [16]. Molecular Breeding Knowledgebase further integrates multiple reference genomes, population sequencing, known alleles, phenotypes, gene expression, and online donor searches [17], while Rice Pan-genome Browser extends allele mining to gene PAV outside a single-reference framework [101]. Interoperable standards such as the Breeding Application Programming Interface can connect germplasm, genotype, phenotype, trial, and marker records across institutional breeding systems [19]. A versioned registry would permit evidence-tier updates without changing the identity of the underlying genetic configuration.

## 5. Future Directions

First, the rice community should establish a public, versioned registry that applies the three-tier hierarchy and records sequence identity, effect estimates, validation results, donor access, assays, and breeding use [15,16,17,97,101]. Failed validation and unsuccessful assay conversion should also be recorded to prevent repeated expenditure, while existing rice databases and BrAPI can provide the technical foundation. Second, discovery should prioritize underrepresented *indica*, *japonica*, *aus*, aromatic, African rice, American breeding material, and wild *Oryza* accessions with reliable phenotypes, available seed, and target-trait relevance [7,8,9,20]. Candidate pan-haplotypes should undergo reciprocal allele replacement, promoter modification, complementation, controlled recombination, NIL development, or targeted editing [28]. Separate tests of the complete haplotype and its component variants can distinguish causal sites from neutral or unfavorable linked alleles.

The third priority should establish product-specific validation across genetic backgrounds and target environments, since favorable performance within one diversity panel does not demonstrate transferability to elite breeding material. Validation should begin with a target product profile that defines the production system, maturity range, grain type, quality requirements, stress exposure, disease pressure, and acceptable agronomic performance, while experimental designs should assess H×B, H×E, dominance, epistasis, pleiotropy, linkage drag, and non-stress performance. A hypothetical package for high yield, premium grain quality, and blast resistance could combine a locally suitable *Ghd7* configuration for maturity and yield potential [47], an *IPA1* configuration for plant architecture [45], a defined *Wx-SSIII-2* profile for eating and cooking quality [67], the validated *Chalk9*-L configuration for reduced chalkiness [75], and the linked PigmR-PigmS receptor pair for blast resistance [93]. This package represents an experimental template and has no presumed compatibility across all rice populations, while factorial or staged evaluation should identify antagonistic effects, redundant components, and undesirable donor segments [24]. Validated major-effect configurations can be fixed through diagnostic markers, while the remaining yield and adaptation background can be improved through genomic prediction [21]. Haplotype-aware prediction should retain an SNP-based comparison model and should enter routine selection only when prospective evaluation demonstrates higher accuracy, lower cost, shorter cycle duration, or greater genetic gain per year [41].

The fourth priority should convert Validated configurations into affordable selection tools and evaluate their operational value within active breeding programs. Each program should specify a comparator, such as phenotypic selection, causal-marker selection, marker-assisted backcrossing, or SNP-based genomic selection, and should measure selection accuracy, genotyping cost, cost per advanced line, family size, assay failure, repeated analysis, field expenditure, cycle duration, realized genetic gain per year, advancement rate, and cultivar-release outcome. KASP assays are appropriate for a limited number of validated large-effect targets, w×ile multiplex amplicon panels, SNP arrays, and low-pass sequencing can support larger packages, recombinant selection, and recipient-genome recovery [18,98,99,100]. National breeding systems can reduce duplicated expenditure through shared genotyping services, version-controlled assay catalogues, common quality-control materials, coordinated donor access, regional trial networks, and interoperable data systems. Implementation can begin with evidence standards, trait-gap lists, and low-cost assay development, advance to causal and multi-environment validation, and culminate in prospective comparisons within national and international breeding pipelines. Success should be measured through stable performance in defined target environments, released pre-breeding lines or cultivars, realized genetic gain, deployment cost, and cycle-time reduction, not through the number of reported haplotypes.

## 6. Conclusions

Haplotype analysis can organize phased genetic variation and identify donors for yield, quality, nutrition, and stress adaptation, but breeding value depends on reproducible effects in elite material and acceptable agronomic performance. The Candidate, Validated, and Breeder-Ready hierarchy separates association signals, genetically supported pre-breeding targets, and configurations suitable for routine selection. Progress requires sequence-defined nomenclature, pangenome resources, causal tests, H×B and H×E evaluation, affordable assays, and prospective breeding comparisons. Haplotype information should complement causal markers, phenotypic selection, and genomic prediction, with success measured through target-environment performance, annual genetic gain, cost, cycle duration, and cultivar delivery.

## Figures and Tables

**Figure 1:**
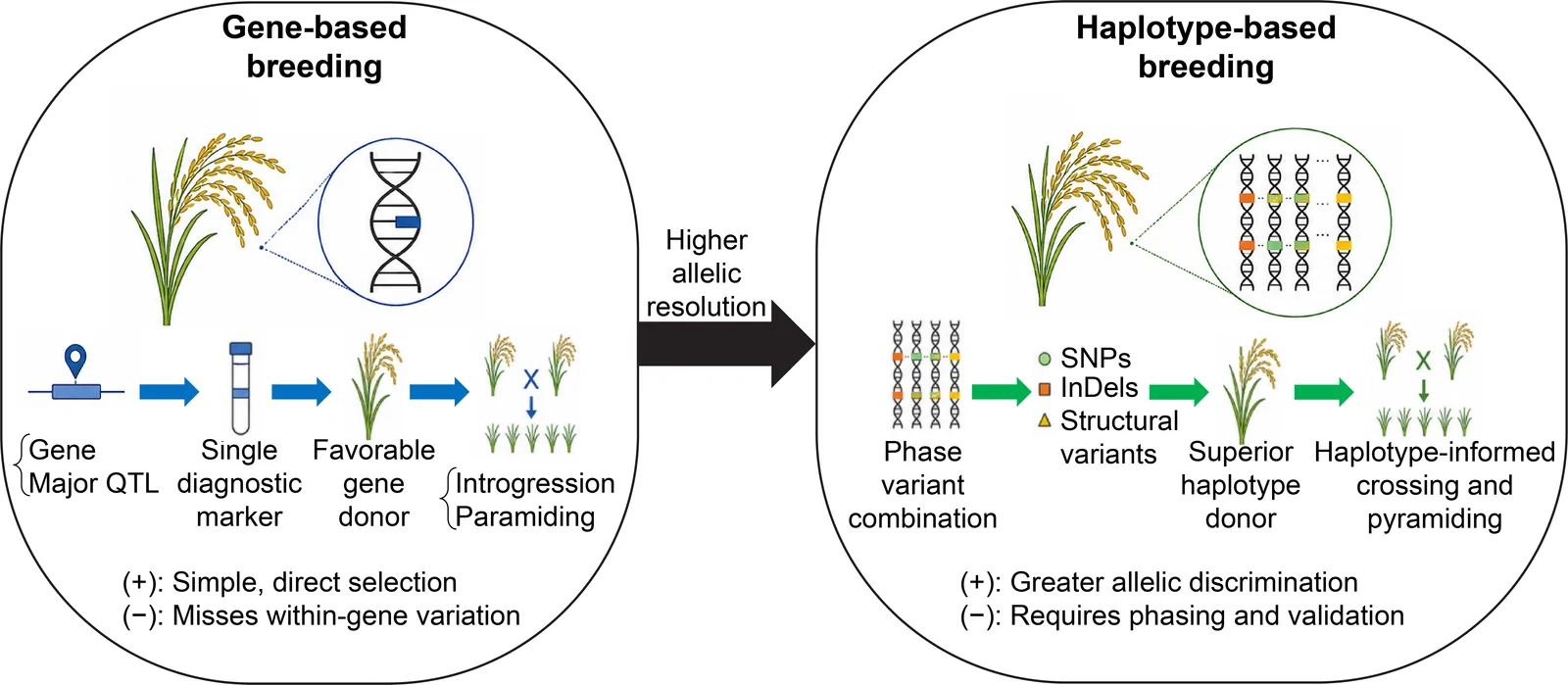
Conceptual comparison of gene-based and haplotype-based breeding in rice. Blue and green arrows indicate the successive steps in the gene-based and haplotype-based workflows, respectively; the black arrow indicates the transition toward higher allelic resolution.

**Figure 2:**
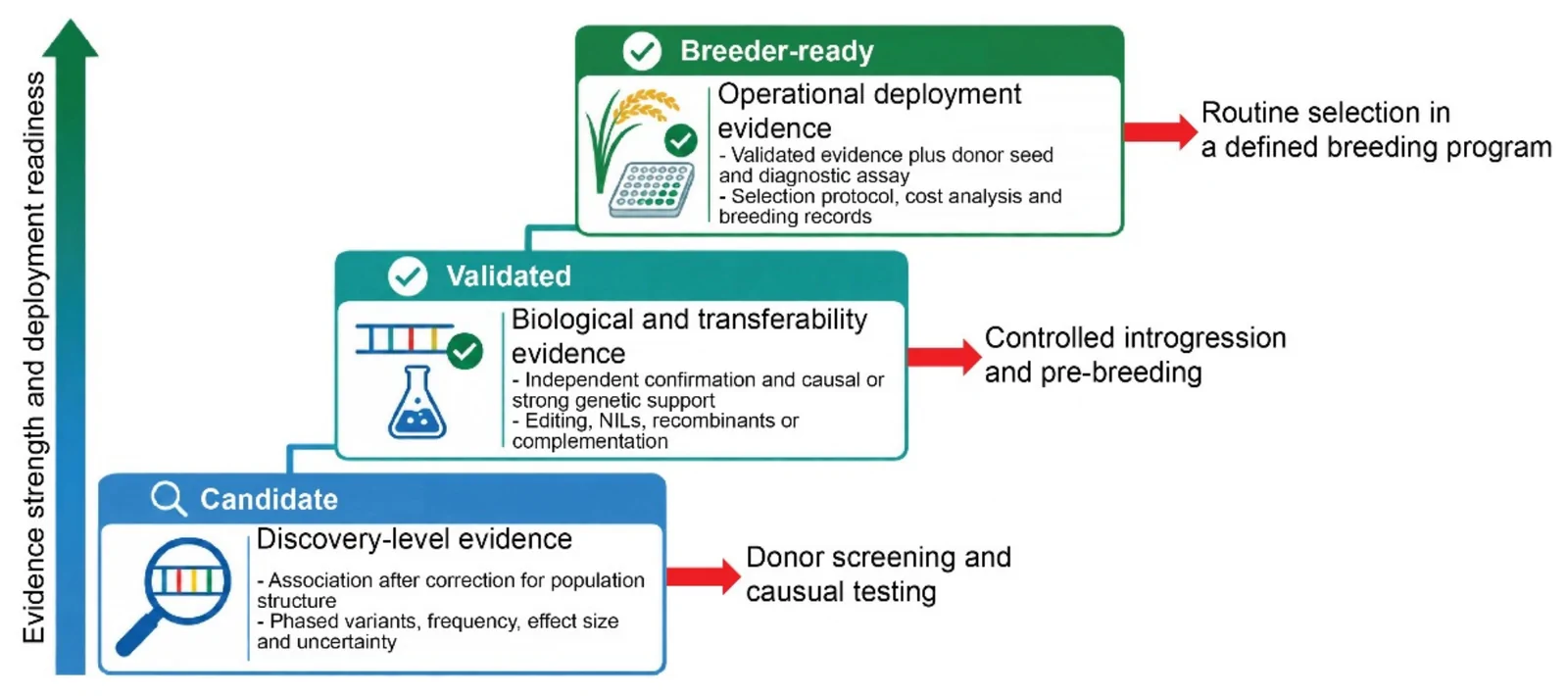
Tiered evidence pathway from haplotype discovery to routine deployment in rice breeding.

**Table 1:** Evidence-ranked genetic configurations associated with yield, plant architecture, grain production, and flowering adaptation in rice.

Breeding Trait	Gene, Locus, or Package	Reported Genetic Configuration	Reported Phenotype or Effect	Population, Frequency, or Donor	Evaluation of H×B, H×E, and Transferability	Evidence Tier	Reference
Plant height	*SD1*	H8	Semi-dwarf stature	30.13% of the surveyed panel	Association across two seasons, with no controlled comparison across elite genetic backgrounds	Candidate	[10]
Tiller number	*MOC1* and *IPA1*	*MOC1*-H9 and *IPA1*-H14	Greater productive tiller number	23.08% for *MOC1*-H9 and 6.64% for *IPA1*-H14	Two-season association, while haplotype effects across *indica*, *japonica*, *aus*, and aromatic groups require controlled testing	Candidate	[10]
Panicle length	*DEP3*, *DEP1*, and *SP1*	H2, H2, and H3	Longer panicles	5.59%, 37.53%, and 5.05%, respectively	Two-season association, with limited information on population-specific LD and architectural trade-offs	Candidate	[10]
Panicle branching	*LAX1*, *LP*, and *OSH1*	H5, H13, and H4	Greater primary branch number	1.56%, 3.64%, and 5.52%, respectively	Low carrier frequencies restrict effect estimation, while validation in elite backgrounds remains unavailable	Candidate	[10]
Grain yield	*PHD1*, *AGO7*, and *ROC5*	H14, H15, and H2	Higher grain yield per plant	15.21%, 3.33%, and 31.42%, respectively	Two-season association without NIL, recombinant, or edited-line confirmation	Candidate	[10]
Early flowering	*Ghd7*	H8, H14, and H6	Earlier flowering, intermediate flowering, and longer growth duration	3.08%, 42.60%, and 1.65%, respectively	Strong sensitivity to photoperiod, latitude, and genetic background requires location-specific evaluation	Candidate	[10,13,47]
Flowering and crop duration	*TOB1*, *TRX1*, *OsVIL3*, and *SNB*	H10, H9, H14, and H9	Early, intermediate, or late flowering according to the reported locus	4.60%, 27.97%, 1.72%, and 9.35%, respectively	Two-season association without controlled photoperiod or multi-latitude trials	Candidate	[10]
Thousand-grain weight	*AET1*, *SNAC1*, *qTGW3*, *DHD1*, and *GW5*	H2, H1, H1, H2, and H1	Higher thousand-grain weight, with reported PVE values of 15.01%, 14.78%, 13.48%, 10.78%, and 5.88%	Carrier frequencies were not reported	Candidate-gene associations require confirmation of causal variants, phase, and effects in additional populations	Candidate	[48]
Thousand-grain weight	Four-gene package	TGW-HC4, composed of *SNAC1*-H1, *qTGW3*-H1, *AET1*-H2, and *DHD1*-H2	Mean thousand-grain weight of 29.47 g	12 of 180 accessions, equivalent to 6.67%	Marker and pedigree support is available, although recombination, epistasis, elite-background performance, and multi-location stability remain untested	Candidate, advanced	[48]
Grain number	*TUT1*	Hap1, Hap2, and Hap4	Grain numbers were 65.1%, 76.1%, and 68.3% higher than those of Hap3	266-accession panel	Three-year GWAS support is available, while controlled introgression and causal reconstruction remain necessary	Candidate	[49]
Grain number and heading date	*Ghd7* and *DTH7*	*Ghd7*-Hap1 and *DTH7*-Hap4	Greater grain number in *indica* for *Ghd7*-Hap1 and in *indica* and *japonica* for *DTH7*-Hap4	216 accessions for *Ghd7* and 266 accessions for *DTH7*	Subgroup-dependent effects support H×B assessment, while maturity effects require evaluation across target production regions	Candidate	[49]
Grain number	*RGN1a*	Hap1 and Hap2	Greater grain number than Hap3 in *japonica*	17 of 95 *japonica* accessions carried the reported favorable class	GWAS and mutant evidence support the locus, although haplotype-specific causality has not been reconstructed	Candidate, supported	[49]
Grain number	*RGN1a-DTH7* package	H1, favorable configuration at both loci	Highest grain number among the reported combinations	82 *japonica* accessions	Joint association does not establish epistasis, while recombination and maturity penalties require controlled evaluation	Candidate	[49]
Panicle length and productivity	*LOC_Os01g43700*, *LOC_Os09g25784*, and *LOC_Os04g47890*	HapA, HapB-HapD, and HapA	Panicle lengths of 27.0 cm or 26.0–27.9 cm, with greater filled-grain number and panicle weight at LOC_Os04g47890	162-accession panel, with *LOC_Os01g43700*-HapA detected in 120 accessions	Three-year GWAS support is available, although causal genes, diagnostic variants, and elite-background effects remain unresolved	Candidate	[50]
Spikelet and grain number	*LOC_Os06g38850*, *LOC_Os06g39050*, and *LOC_Os04g11040*	HapD, HapA, and HapA	Mean total spikelet number of 194.0 and 172.53 versus 153.34 spikelets, and 120.95 versus 110.76 grains	Carrier numbers of 8, 160, and 45, respectively	Two-environment GWAS support is available, while the low carrier number for LOC_Os06g38850 limits effect precision	Candidate	[51]
Grain filling	*LOC_Os03g62720*, *LOC_Os03g62750*, *LOC_Os03g62820*, *LOC_Os12g03450*, and *LOC_Os12g03470*	HapA, HapA or HapB, HapC, HapA, and HapA	Reported grain-filling percentages ranged from 70.9% to 79.1% for the favorable classes	Carrier numbers ranged from 11 to 140	Two-environment GWAS support requires independent confirmation under heat, drought, and contrasting crop-management conditions	Candidate	[51]
Grain yield	*EPN4-GNP1* package	*EPN4*-1 plus *GNP1*-6	Higher grain yield than alternative configurations	Frequency not reported	Joint-haplotype analysis supports a testable package, although additive and non-additive effects require recombinant or edited materials	Candidate	[52]
Panicle exsertion	*EPE1/OsGA2ox9* and *EUI1*	*EPE1*-Hap12 and *EUI1*-HapB	Panicle exsertion length of 7.7 cm for *EPE1*-Hap12 and a positive effect of 0.23 cm for *EUI1*-HapB	*EPE1*-Hap12 occurred in 31 accessions from the 3K panel, while *EUI1*-HapB was examined in a 440-accession panel	Association evidence requires controlled evaluation of internode elongation, lodging, fertility, and background dependence	Candidate	[53]
Grain size and filling	*OsSWEET11* and *OsSWEET15*	Study-defined superior haplotypes	Variation in grain size and grain weight associated with sucrose transport during grain filling	139 Yunnan rice landraces	Geographic restriction and local population structure limit direct transfer to major elite *indica* and *japonica* pools	Candidate	[54]
Grain length diversity	*GS3*	GS3-H4	Ancestral haplotype associated with grain-shape diversity	Predominantly wild rice, with occurrence in several *aus* and aromatic accessions	Distribution identifies potential donors but does not demonstrate favorable performance in elite backgrounds	Candidate	[55]
Plant height and lodging resistance	*sd1*	Defective *GA20ox-2* allele	Reduced plant height and improved lodging resistance associated with Green Revolution cultivars	Deployed across numerous semi-dwarf rice backgrounds	Causal evidence and historical breeding use are strong, although the cited studies do not provide the complete assay, cost, and current pipeline records required by the Breeder-Ready tier	Validated, deployment benchmark	[46]
Grain number	*Gn1a/OsCKX2*	Reduced-expression or loss-of-function allele derived from Habataki	Increased cytokinin concentration in inflorescence meristems and greater grain number	Initially characterized through *indica*-*japonica* mapping materials	Map-based cloning and controlled genetic evidence support causality, while yield advantage depends on assimilate supply and recipient background	Validated	[49,56]
Panicle architecture	*DEP1*	Truncated favorable allele	Dense erect panicles, greater branch number, increased grain number, and improved nitrogen response in tested materials	Common in several erect-panicle *japonica* cultivars	Functional and genetic evidence supports the allele, although canopy density, grain filling, and regional adaptation require product-specific testing	Validated	[57]
Tiller number, panicle branching, and culm strength	*IPA1/OsSPL14*	Increased OsSPL14 activity associated with altered miR156 regulation	Fewer unproductive tillers, greater panicle branching, stronger culms, and improved lodging resistance	Characterized in *indica* and *japonica* materials	Functional evidence supports the architectural effect, while optimal expression depends on genetic background and crop-management conditions	Validated	[45]
Grain length and weight	*GS3*	Loss-of-function allele	Increased grain length and altered grain weight	Characterized through mapping populations and edited materials	Causal evidence is available, although market-class requirements and genetic-background effects determine breeding value	Validated	[23,58,59]
Grain width and weight	*GW2*	Reduced-function favorable allele	Increased grain width and weight through altered cell proliferation	Mapping and functional materials from the cited study	Functional evidence supports causality, while pleiotropic effects on grain shape and quality require recipient-specific evaluation	Validated	[10,60]
Grain width and yield	*GW5/qSW5*	Domestication-associated deletion or favorable allele	Increased grain width and grain weight	Frequent in cultivated germplasm selected for wider grains	Map-based and functional evidence supports the locus, although consumer preference and linked quality traits constrain deployment	Validated	[48,61,62]
Heading date and yield potential	*Ghd7* and *Ghd8*	Functional allelic classes	Changes in heading date, plant height, grain number, and regional adaptation	Allele distributions differ among rice subgroups and production regions	Strong photoperiod sensitivity produces major H×E effects, while interaction with *Hd1*, *OsPRR37*, and other flowering loci produces H×B effects	Validated, environment-dependent	[13,47]
Grain yield balance	*EPN4/NAL1*	*EPN4*-1	Balanced effective panicle number, grain number, and grain weight, with the highest yield among seven compared haplotypes	Seven haplotypes examined in association and NIL materials	NIL evidence supports the locus, while transfer to additional elite backgrounds and target environments remains necessary	Validated	[52]
Grain number and weight	*MOG1*	Hap-HNW	Greater grain number and heavier grains	Present in approximately 96% of the examined upland accessions	Map cloning and functional assays support the genetic effect, while its high frequency in upland rice may limit additional selection gain within that group	Validated	[63]
Grain number and panicle branching	*GNP3/OsMKKK22*	Hap-T	Greater grain number and panicle branch number	Prevalent in *indica* germplasm	NIL and functional evidence support the locus, while grain filling and performance in *japonica* backgrounds require further testing	Validated	[64]
Grain number	*GNP2/OsGSK1* and *GNP5*	*GNP2*-Hap1 or Hap2 and *GNP5*-Hap1	Greater grain number than the alternative haplotypes	*GNP2* classes occurred in 496 and 657 accessions, while *GNP5*-Hap1 occurred in 496 accessions	Complementation, functional assays, and tests at two sites support the loci, while recipient-background effects remain relevant	Validated	[65]
Grain number and yield	*GNP2-GNP5* package	Type I	Highest grain number and grain yield among the reported combinations	20.2% of *japonica* accessions	Two-site association and NIL evidence support the configuration, although formal epistasis tests and multi-region trials remain necessary	Validated, conditional package	[65]
Culm strength, architecture, and yield	*STRONG1*	Hap-STRONG1C	Stronger culms, improved plant and panicle architecture, and higher yield	Identified in a 795-accession panel	NIL and field evidence support the haplotype, while diagnostic marker conversion and trials in additional elite recipients remain necessary	Validated	[66]
Panicle exsertion	*EPE1/OsGA2ox9*	HapD	Mean panicle exsertion length of 6.73 cm in *japonica*	440-accession panel	Two-year GWAS and CRISPR evidence support the locus, while excessive internode elongation and lodging risk require product-specific assessment	Validated	[53]
Grain number and weight package	*gn1a*, *gs3*, and *ipa1*	*gn1a-gs3*, *gs3-ipa1*, and *gn1a-gs3-ipa1* combinations	Greater yield per plant or heavier grains than corresponding single mutants, with increased plant height in several combinations	Edited Zhonghua 11 *japonica* background	Controlled genetic evidence demonstrates non-additive outcomes and an architectural penalty, while additional backgrounds and locations remain necessary	Validated, background-limited	[23]
Grain shape for a regional market class	*GS3* and *qGL7.1*	Regionally validated haplotype classes and linked diagnostic markers	Grain-length effects suited to U.S. long-grain and medium-grain breeding materials	323 elite U.S. rice genotypes with biparental validation	Regional validation and KASP marker development support applied breeding use, although transfer beyond the tested market classes requires new validation	Validated, regional marker-ready	[30]

**Table 2:** Evidence-ranked genetic configurations associated with grain appearance, eating and cooking quality, aroma, protein composition, mineral concentration, predicted glycemic index, grain oil, and antioxidant traits in rice.

Quality Category	Gene or Locus	Reported Genetic Configuration	Reported Phenotype	Population, Frequency, or Donor	Evaluation of H×B, H×E, and Transferability	Evidence Tier	Reference
Heat-stable appearance and milling quality	*GBSSI/Wx-SSIIa/ALK* package	GGAAC-GGC	Low chalkiness and high head-rice recovery under high temperature	Seven accessions with the lowest chalkiness scores within a 60-accession panel	Evaluated across two hot-season field trials at one location, while genetic-background effects and performance across additional heat-prone regions require testing	Candidate	[42]
Amylose content	*RSR1*	H8	Intermediate amylose content	4.20% of the 3K panel	Two-season association without controlled introgression or causal-variant confirmation	Candidate	[10]
Amylose content and texture	*Wx*	TT-AA-CC	Amylose content of 6.09% and gel consistency of 114.08 mm	Segregating population	Physicochemical evidence supports soft cooked rice, while market acceptance and effects across *indica* and *japonica* backgrounds require evaluation	Candidate	[67]
Amylose content and texture	*Wx*	TT-AA-TT	Amylose content of 8.03% and gel consistency of 111.87 mm	Segregating population	Association with soft texture requires confirmation under different grain-filling temperatures and nitrogen regimes	Candidate	[67]
Amylose content	*Os06g0232700*	H4 and H5	Amylose contents of 26.50% and 24.00%, respectively	H5 occurred in 57.33% of a 150-accession 3K subset	GWAS and haplotype-phenotype evidence without functional confirmation of the reported variant classes	Candidate	[68]
Amylose content	*Os06g18710*	H3 and H6	Amylose contents of 25.50% and 23.30%, respectively	150-accession 3K subset	Carrier frequencies and independent background tests were not reported	Candidate	[68]
Amylose content	*Os06g18720*	H4 and H14	Amylose contents of 26.58% and 23.36%, respectively	150-accession 3K subset	Population structure and LD may influence the reported associations, while causal variants remain unresolved	Candidate	[68]
Gelatinization temperature	*Os06g0229000* and *Os06g12380*	Os06g0229000-H4 and Os06g12380-H5	Intermediate ASV values of 3.67 and 4.48, respectively	Os06g12380-H5 occurred in 41.33% of the tested panel	Association evidence requires cooking tests and evaluation across market classes with different gelatinization preferences	Candidate	[68]
Gel consistency	*Os10g046900*	H1	Gel consistency of 56.06 mm	150-accession 3K subset	The reported value requires confirmation in elite materials with defined amylose and gelatinization profiles	Candidate	[68]
Resistant starch	*Os06g11100*	H4 and H2	Resistant starch contents of 3.28% and 2.42%, respectively	150-accession 3K subset	Association and physicochemical evidence without independent genetic confirmation	Candidate	[68]
Resistant starch	*Os06g16080*	H11 and H7	Resistant starch contents of 5.37% and 2.95%, respectively	150-accession 3K subset	Effects on cooking time, texture, retrogradation, and consumer acceptance require product-specific testing	Candidate	[68]
Resistant starch	*Os02g16970*	H14	Resistant starch content of 4.49%	150-accession 3K subset	Functional evidence and carrier frequency were not reported	Candidate	[68]
Predicted glycemic index	*Os08g12590*	H13	Lower or intermediate predicted glycemic index of 62.52	150-accession 3K subset	Predicted glycemic index requires confirmation through standardized starch-digestion assays and human nutritional studies	Candidate	[68]
Resistant starch and cooking quality	*Wx-SSIIa* package	*Wx*^a^-*SSIIa*^G-TT^	Higher cooked and retrograded resistant starch with lower gelatinization and retrogradation values	CG133R × Javanica 22 recombinant inbred lines	QTL and recombinant-line evidence supports an advanced Candidate package, while additional backgrounds, processing methods, and sensory panels require evaluation	Candidate, advanced	[69]
Grain iron and zinc	*OsNAS3*	H2	Higher iron and zinc concentrations	30 accessions and 1.00% of the 3K panel	Low frequency identifies a rare donor, while controlled introgression and assessment of cadmium accumulation remain necessary	Candidate	[10]
Grain iron and zinc	*Os06g0489500*	Hap3	Higher iron and zinc without a detected increase in cadmium	265 *indica* or Xian accessions	The association requires confirmation in elite *indica* materials across soils with contrasting mineral availability	Candidate	[70]
Grain iron and zinc	*Os07g0568300*	Hap4, Hap8, and Hap6	Hap4 and Hap8 increased zinc, while Hap6 increased iron without detected cadmium effects	265 *indica* accessions for zinc and 433 *japonica* accessions for iron	Subgroup-dependent effects provide evidence of H×B and require separate validation in *indica* and *japonica* breeding pools	Candidate	[70]
Grain zinc and oil	Zinc- and oil-associated blocks	ACCC and CT	Higher grain zinc concentration for ACCC and higher grain oil content for CT	184 deepwater rice accessions from Assam evaluated for two years	Geographic restriction and local population structure limit transfer to elite irrigated rice without controlled crossing and validation	Candidate	[71]
Grain lysine	Study-defined QTNs and haplotype classes	Favorable alleles identified through multi-model GWAS	Higher lysine accumulation	387 rice accessions	QTN-by-environment effects demonstrate environmental sensitivity, while diagnostic variants and elite-background performance remain unresolved	Candidate	[72]
Phenolic content and antioxidant capacity	*Rc*	H002	Higher phenolic content and antioxidant activity	84 Shanlan upland rice landraces	Association evidence requires confirmation of pigment stability, cooking retention, yield, and grain-quality effects	Candidate	[73]
Black pericarp	*OsKala4*	H01	Functional black-pericarp pigmentation	95.9% of the examined black rice accessions	Population-genomic evidence supports the haplotype distribution, while the specific H01 effect requires controlled genetic comparison	Candidate	[74]
Seed anthocyanin content	*OsMYB3*	H01	Higher seed anthocyanin concentration	79.3% of the examined black rice accessions	Multi-omics and association evidence requires confirmation across genetic backgrounds, environments, storage periods, and cooking methods	Candidate	[74]
Grain chalkiness	*Chalk9*	*Chalk9*-L with a causal 64-bp promoter insertion	Higher *Chalk9* expression, lower chalky grain rate, and lower degree of chalkiness without a detected yield penalty	Present in all 123 surveyed *japonica* cultivars and 81% of 105 recent elite *indica* cultivars	GWAS, NIL, transgenic, promoter-editing, and yield evidence support causality, while performance under high grain-filling temperatures requires multi-environment assessment	Validated, marker prototype available	[75]
Grain chalkiness and shape	*Chalk7-GS3-Chalk5* package	*Chalk7*^Cyp^, *GS3*^MH^, and *Chalk5*^MH^	More slender grains and a major reduction in chalkiness without a detected yield penalty	Rare in natural germplasm and evaluated through NILs in a common elite background	Three allele-specific markers and one- to three-locus NILs support the package, while evaluation across additional elite backgrounds and production regions remains necessary	Validated, pre-breeding package	[59]
Taste quality	*OsGATA7*	Hap1	Lower grain protein content and higher taste score through reduced *OsGluA2* expression	Evaluated within a 2280-accession panel	Association, CRISPR, and introgression evidence support the locus, while nitrogen response and sensory performance across market classes require further testing	Validated	[76]
Taste quality	*SMOS1*	Hap1	Lower protein accumulation and improved taste quality	Detected mainly in *japonica* germplasm	Association and introgression-line evidence support the effect, while transfer to *indica* backgrounds may alter protein composition and cooked-rice texture	Validated, background-sensitive	[76]
Aroma	*BADH2/fgr*	Hap6	Fragrant grain with accumulation of 2-acetyl-1-pyrroline	5.8% of the 3K panel and approximately 51% of the aromatic subgroup, with regional testing in U.S. rice	Global haplotype analysis, aroma assays, and KASP validation support marker-assisted use, while deployment should retain the required grain shape, maturity, and cooking quality	Validated, regional marker-ready	[29]
Grain protein and amino acids	*OsAAP6/qPC1*	Group A, types 1–5	Higher grain protein and total amino acid contents	197-accession mini-core collection, with greater representation in *indica*	NIL and transgenic evidence support causality, while eating quality and nitrogen-management responses require assessment	Validated	[77]
Grain protein and glutelin	*OsGluA2/qGPC-10*	*OsGluA2* ^HET^	Higher glutelin and total grain protein contents	Predominant in *indica* within a 3005-accession panel	QTL cloning, CRISPR, and population evidence support the allele, while taste, texture, and nutritional value require product-specific evaluation	Validated	[78]
Regional grain shape	*GS3* and *qGL7.1*	*GS3*-Hap1 or Hap3 and marker-defined *qGL7.1* classes	*GS3*-Hap1 had a mean grain length of 7.13 mm, while Hap3 had a mean of 5.94 mm and differentiated U.S. long- and medium-grain classes	323 elite U.S. rice genotypes with biparental recombinant-line validation	Functional and linked KASP markers were validated for an applied U.S. breeding program, while transfer to other market classes requires new regional testing	Validated, regional marker-ready	[30]

**Table 3:** Evidence-ranked genetic configurations associated with abiotic-stress adaptation and biotic resistance in rice.

Stress or Constraint	gene or Locus	Reported Genetic Configuration	Reported Phenotype	Population, Frequency, or Donor	Evaluation of H×B, H×E, and Transferability	Evidence Tier	Reference
Drought	*OsGSK1*	H4	Higher spikelet yield under drought than other haplotype classes	Approximately 47% of a 399-accession *indica*-*aus* panel	The association requires confirmation in contrasting elite backgrounds and managed drought environments	Candidate	[79]
Drought	*OsDSR2*	H3	Favorable drought-response phenotype within the discovery panel	Approximately 13% of the 399-accession panel	Limited population frequency and the absence of reciprocal near-isogenic comparisons restrict transferability	Candidate	[79]
Drought	*OsDIL1*, *OsDREB1C*, *DSM3*, and *ZFP182*	H22, H3, H4, and H4, respectively	Superior drought-response traits within the association panel	A 399-accession *indica*-*aus* panel, with *OsDREB1C*-H3 also detected in five drought-tolerant varieties	Background effects and environment-dependent drought responses require multi-parent and multi-location evaluation	Candidate	[79]
Drought	*ASR3*	H88	Favorable drought-response phenotype in the discovery analysis	Three accessions among 399 accessions, equivalent to approximately 0.75%	Very low frequency limits effect estimation and demands targeted donor validation before introgression	Candidate	[79]
Salinity	*OsSTL1*	Hap1	Greater seedling salt tolerance associated with the favorable locus class	A 664-accession panel, with the favorable lead allele detected in 98.5% of *indica* and 23.0% of *japonica* accessions	Strong subspecies differentiation indicates a high probability of H×B effects after transfer between *indica* and *japonica*	Candidate	[80]
Salinity	*OsSTL2*	Hap1 and Hap2	Improved seedling salt-tolerance measurements within favorable haplotype classes	A 664-accession panel, with the favorable lead allele detected in 99.3% of *indica* and 78.0% of *japonica* accessions	High allele frequency may reduce selection gain in *indica*, while haplotype effects require evaluation beyond the seedling stage	Candidate	[80]
Salinity	*OsSTL1-OsSTL2*	Co-hap2, defined as *OsSTL1*-Hap1 plus *OsSTL2*-Hap2	Ten of the eleven accessions with the highest salt tolerance carried this configuration	Derived from the 664-accession association panel	This multilocus package is not one physical haplotype, and its value requires phased marker analysis and progeny-based testing	Candidate	[80]
Salinity	*BADH1*	Hap_2, Hap_18, and Hap_23 as the major tested classes	Haplotype-associated variation in germination percentage and germination index under salt treatment, with Hap_18 associated with salt sensitivity	Thirty-nine haplotypes identified across 475 rice accessions	Evidence is restricted to germination-stage phenotypes, while reproductive-stage effects and H×B responses remain untested	Candidate	[81]
Salinity	*OsCUL3c*	Salt-response-associated haplotype identified within the local Thai collection	Differences in seed germination, shoot growth, and physiological responses under salt treatment	A collection of 233 Thai rice accessions with 79 reported haplotypes	Extensive local diversity supports donor discovery, although transfer to non-Thai germplasm requires haplotype reconstruction and functional testing	Candidate	[82]
Low phosphorus	*OsAAD*	Hap1	Higher shoot dry weight and grain yield under normal and low-phosphorus supply than the other main haplotypes	Four main haplotypes identified among 191 accessions, with Hap1 concentrated in *indica* germplasm	Two-year field data support the association, although the causal natural variant within Hap1 has not received haplotype-specific validation	Candidate	[83]
Iron toxicity	Candidate genes within iron-toxicity-associated loci	Study-defined favorable haplotypes	Lower leaf bronzing and improved seedling performance under excess iron	A diverse panel of 551 rice accessions	Complex iron responses and strong environment effects require validation in acidic lowland soils across developmental stages	Candidate	[84]
Anaerobic germination	*OsTPP7*	Hap_1	Greater coleoptile elongation and seedling establishment under flooded germination conditions	Detected in 395 of 475 accessions and concentrated mainly in *japonica*	High frequency within *japonica* limits within-group selection, while transfer into *indica* requires background-specific evaluation	Candidate	[85]
Blast resistance	*LOC_Os12g39700*	H4	Lower blast disease scores within the association subset	A 147-accession subset with representation in *aus* and *indica*	The small discovery subset and pathogen-isolate dependence restrict immediate use across breeding populations	Candidate	[86]
Blast resistance	*LOC_Os06g30440* or *OsGH3-7*	H33	Favorable blast-response phenotype within the association subset	A 147-accession subset	Validation with defined pathogen isolates and near-isogenic materials is required before marker deployment	Candidate	[86]
Bacterial leaf blight	*LOC_Os02g12660* or *RLCK68*	H39	Lower disease severity within the *aus* group	A 147-accession subset with the favorable class concentrated in *aus*	Subpopulation restriction indicates possible H×B effects and limited immediate transfer to *indica* or *japonica* breeding pools	Candidate	[86]
Bacterial leaf blight	*LOC_Os05g35490*	H49	Favorable bacterial-blight response within the *indica* group	A 147-accession subset	Pathogen-race specificity and the limited validation population constrain selection outside the discovery material	Candidate	[86]
Drought	*DROT1*	Hap3, associated with the promoter C-to-T variant at s18975900	Greater drought resistance through improved root growth and water acquisition	Identified across 743 accessions and enriched in upland tropical *japonica* and *indica* germplasm	Functional evidence supports the allele, while performance across irrigated and rainfed elite backgrounds requires additional evaluation	Validated	[87]
Drought	*DRG9*	Hap1	Improved drought resistance and grain yield under water limitation	Detected in 130 of 503 accessions and introduced into Huanghuazhan	Introgression into an elite recipient supports breeding value, although additional target environments and recipient groups remain necessary	Validated	[88]
Salinity	*OsWRKY53*	Haplotype A	Improved salt tolerance through regulation of ion balance and stress-response pathways	Identified through landrace-based association analysis and subsequent functional experiments	Functional support strengthens causal inference, while evaluation remains concentrated in limited backgrounds and growth stages	Validated	[89]
Heat	*SLG1*	Hap2 or *SLG1-Ind*	Higher survival and improved growth under high temperature, with reciprocal near-isogenic lines showing approximately 81% survival compared with 48%	Detected in 2527 of 2691 *indica* accessions, equivalent to approximately 93.9%	Reciprocal near-isogenic evidence supports the effect, although the high *indica* frequency limits selection gain within this subspecies	Validated	[90]
Heat	*TTL1*	HapL	Improved thermotolerance, pollen performance, and yield-related traits under heat stress	Identified within a 187-accession core panel and concentrated mainly in *indica*	Functional support is available, while multi-location reproductive-stage evaluation is needed for regional deployment	Validated	[91]
Cold at booting	*CTB3*	Hap1	Improved cold tolerance, spikelet fertility, and grain set during the booting stage	Identified in a 525-accession panel and concentrated mainly in *japonica*	Subspecies concentration indicates restricted transferability, particularly when Hap1 is introduced into *indica* recipients	Validated	[92]
Cold at booting	*OsTCP19-CTB3*	Group I, defined as *OsTCP19*-T-Hap1 plus *CTB3*-Hap1	Greater cold tolerance than alternative two-locus groups	Reported only within *japonica* germplasm	The configuration represents a two-locus package, and recombination, phase, and recipient-background effects require progeny-based assessment	Validated	[92]
Blast resistance	*Pigm*	Linked PigmR-PigmS receptor configuration	Resistance to multiple blast isolates with reduced yield penalty compared with unbalanced resistance-gene expression	Derived from the donor Gumei 4	The linked receptor pair should be transferred as a phased interval, while local pathogen populations require disease screening	Validated	[93]
Brown planthopper	*BPH1/9*	*BPH1/9-7* and *BPH1/9-9*	Strong brown-planthopper resistance associated with functional allelic variation	Twenty-one haplotypes detected among 117 varieties, with T12 and Pokkali serving as reported donors	Donor validation supports introgression, although resistance durability requires testing against regional insect populations	Validated	[94]
Rice stripe virus	*STV11*	R and R′ resistance classes	Reduced viral multiplication and improved resistance to rice stripe virus	Evaluated across 271 cultivated accessions and 30 wild accessions, with resistant classes detected in 85.03% of *indica* accessions	High *indica* frequency and vector-population differences should guide parent choice and regional evaluation	Validated	[95]
Submergence	*Sub1A*	Functional *Sub1A-1* allele, also reported as H8 within one panel-based classification	Survival after complete submergence through restricted elongation and improved carbohydrate conservation	Derived from FR13A and deployed in several high-yielding rice backgrounds	Diagnostic markers, repeated introgression, field evaluation, and cultivar deployment support direct breeding use, although flooding duration and water conditions remain relevant	Breeder-Ready	[2,26,96]
Bacterial leaf blight	*xa5*, *xa13*, and *Xa21*	Three-gene resistance package	Resistance to several bacterial-blight races with retention of the preferred agronomic background	Deployed in improved versions of PR106 and Samba Mahsuri	This configuration comprises three unlinked resistance genes and is not one haplotype, while diagnostic markers and cultivar-level deployment support operational use	Breeder-Ready	[34,35]

## Data Availability

No new datasets were generated or analyzed in this review. All data discussed in this article are available from the cited references.

## References

[1] Jena K.K., Mackill D.J. (2008). Molecular markers and their use in marker-assisted selection in rice. Crop Sci..

[2] Xu K., Xu X., Fukao T., Canlas P., Maghirang-Rodriguez R., Heuer S., Ismail A.M., Bailey-Serres J., Ronald P.C., Mackill D.J. (2006). Sub1A is an ethylene-response-factor-like gene that confers submergence tolerance to rice. Nature.

[3] Gamuyao R., Chin J.H., Pariasca-Tanaka J., Pesaresi P., Catausan S., Dalid C., Slamet-Loedin I., Tecson-Mendoza E.M., Wissuwa M., Heuer S. (2012). The protein kinase Pstol1 from traditional rice confers tolerance of phosphorus deficiency. Nature.

[4] Chin J.H., Lu X., Haefele S.M., Gamuyao R., Ismail A., Wissuwa M., Heuer S. (2010). Development and application of gene-based markers for the major rice QTL Phosphorus uptake 1. Theor. Appl. Genet..

[5] Ren Z.-H., Gao J.-P., Li L.-G., Cai X.-L., Huang W., Chao D.-Y., Zhu M.-Z., Wang Z.-Y., Luan S., Lin H.-X. (2005). A rice quantitative trait locus for salt tolerance encodes a sodium transporter. Nat. Genet..

[6] Wang W., Mauleon R., Hu Z., Chebotarov D., Tai S., Wu Z., Li M., Zheng T., Fuentes R.R., Zhang F. (2018). Genomic variation in 3,010 diverse accessions of Asian cultivated rice. Nature.

[7] Fuentes R.R., Chebotarov D., Duitama J., Smith S., De la Hoz J.F., Mohiyuddin M., Wing R.A., McNally K.L., Tatarinova T., Grigoriev A. (2019). Structural variants in 3000 rice genomes. Genome Res..

[8] Shang L., Li X., He H., Yuan Q., Song Y., Wei Z., Lin H., Hu M., Zhao F., Zhang C. (2022). A super pan-genomic landscape of rice. Cell Res..

[9] Guo D., Li Y., Lu H., Zhao Y., Kurata N., Wei X., Wang A., Wang Y., Zhan Q., Fan D. (2025). A pangenome reference of wild and cultivated rice. Nature.

[10] Abbai R., Singh V.K., Nachimuthu V.V., Sinha P., Selvaraj R., Vipparla A.K., Singh A.K., Singh U.M., Varshney R.K., Kumar A. (2019). Haplotype analysis of key genes governing grain yield and quality traits across 3K RG panel reveals scope for the development of tailor-made rice with enhanced genetic gains. Plant Biotechnol. J..

[11] Huang X., Wei X., Sang T., Zhao Q., Feng Q., Zhao Y., Li C., Zhu C., Lu T., Zhang Z. (2010). Genome-wide association studies of 14 agronomic traits in rice landraces. Nat. Genet..

[12] Du M., Xiong M., Chang Y., Liu Z., Wang R., Lin X., Zhou Z., Lu M., Liu C., Liu E. (2022). Mining candidate genes and favorable haplotypes for flag leaf shape in rice (*Oryza sativa* L.) based on a genome-wide association study. Agronomy.

[13] Zhang B., Liu H., Qi F., Zhang Z., Li Q., Han Z., Xing Y. (2019). Genetic interactions among *Ghd7*, *Ghd8*, *OsPRR37* and *Hd1* contribute to large variation in heading date in rice. Rice.

[14] Wei X., Xu J., Guo H., Jiang L., Chen S., Yu C., Zhou Z., Hu P., Zhai H., Wan J. (2010). DTH8 suppresses flowering in rice, influencing plant height and yield potential simultaneously. Plant Physiol..

[15] Mansueto L., Fuentes R.R., Borja F.N., Detras J., Abriol-Santos J.M., Chebotarov D., Sanciangco M., Palis K., Copetti D., Poliakov A. (2017). Rice SNP-seek database update: New SNPs, indels, and queries. Nucleic Acids Res..

[16] Wang C.-C., Yu H., Huang J., Wang W.-S., Faruquee M., Zhang F., Zhao X.-Q., Fu B.-Y., Chen K., Zhang H.-L. (2020). Towards a deeper haplotype mining of complex traits in rice with RFGB v2.0. Plant Biotechnol. J..

[17] Peng H., Wang K., Chen Z., Cao Y., Gao Q., Li Y., Li X., Lu H., Du H., Lu M. (2020). MBKbase for rice: An integrated omics knowledgebase for molecular breeding in rice. Nucleic Acids Res..

[18] Arbelaez J.D., Dwiyanti M.S., Tandayu E., Llantada K., Jarana A., Ignacio J.C., Platten J.D., Cobb J., Rutkoski J.E., Thomson M.J. (2019). 1k-RiCA (1K-Rice Custom Amplicon) a novel genotyping amplicon-based SNP assay for genetics and breeding applications in rice. Rice.

[19] Selby P., Abbeloos R., Backlund J.E., Basterrechea Salido M., Bauchet G., Benites-Alfaro O.E., Birkett C., Calaminos V.C., Carceller P., Cornut G. (2019). BrAPI—An application programming interface for plant breeding applications. Bioinformatics.

[20] Zhao Q., Feng Q., Lu H., Li Y., Wang A., Tian Q., Zhan Q., Lu Y., Zhang L., Huang T. (2018). Pan-genome analysis highlights the extent of genomic variation in cultivated and wild rice. Nat. Genet..

[21] Spindel J., Begum H., Akdemir D., Virk P., Collard B., Redoña E., Atlin G., Jannink J.-L., McCouch S.R. (2015). Genomic selection and association mapping in rice (*Oryza sativa*): Effect of trait genetic architecture, training population composition, marker Number and statistical model on accuracy of rice genomic selection in elite, tropical rice breeding lines. PLoS Genet..

[22] Xu Y., Wang X., Ding X., Zheng X., Yang Z., Xu C., Hu Z. (2018). Genomic selection of agronomic traits in hybrid rice using an NCII population. Rice.

[23] Li M., Pan X., Li H. (2022). Pyramiding of gn1a, gs3, and ipa1 exhibits complementary and additive effects on rice yield. Int. J. Mol. Sci..

[24] Liu X., Deng X., Kong W., Sun T., Li Y. (2023). The pyramiding of elite allelic genes related to grain number increases grain number per panicle using the recombinant lines derived from *indica-japonica* cross in rice. Int. J. Mol. Sci..

[25] Hu Z., Wang W., Wu Z., Sun C., Li M., Lu J., Fu B., Shi J., Xu J., Ruan J. (2018). Novel sequences, structural variations and gene presence variations of Asian cultivated rice. Sci. Data.

[26] Neeraja C.N., Maghirang-Rodriguez R., Pamplona A., Heuer S., Collard B.C.Y., Septiningsih E.M., Vergara G., Sanchez D., Xu K., Ismail A.M. (2007). A marker-assisted backcross approach for developing submergence-tolerant rice cultivars. Theor. Appl. Genet..

[27] Dixit S., Yadaw R.B., Mishra K.K., Kumar A. (2017). Marker-assisted breeding to develop the drought-tolerant version of Sabitri, a popular variety from Nepal. Euphytica.

[28] Thomson M.J., Biswas S., Tsakirpaloglou N., Septiningsih E.M. (2022). Functional allele validation by gene editing to leverage the wealth of genetic resources for crop improvement. Int. J. Mol. Sci..

[29] Addison C.K., Angira B., Kongchum M., Harrell D.L., Baisakh N., Linscombe S.D., Famoso A.N. (2020). Characterization of haplotype diversity in the BADH2 aroma gene development of a KASPSNPassay for predicting aroma in, U.S. rice. Rice.

[30] Angira B., Cerioli T., Famoso A.N. (2022). Discovery validation of grain shape loci in, U.S. rice germplasm through haplotype characterization. Front. Genet..

[31] Ayres N.M., McClung A.M., Larkin P.D., Bligh H.F.J., Jones C.A., Park W.D. (1997). Microsatellites and a single-nucleotide polymorphism differentiate apparent amylose classes in an extended pedigree of US rice germ plasm. Theor. Appl. Genet..

[32] Spielmeyer W., Ellis M.H., Chandler P.M. (2002). Semidwarf (*sd-1*), “green revolution” rice, contains a defective gibberellin 20-oxidase gene. Proc. Natl. Acad. Sci. USA.

[33] Bradbury L.M.T., Henry R.J., Jin Q., Reinke R.F., Waters D.L.E. (2005). A perfect marker for fragrance genotyping in rice. Mol. Breed..

[34] Singh S., Sidhu J.S., Huang N., Vikal Y., Li Z., Brar D.S., Dhaliwal H.S., Khush G.S. (2001). Pyramiding three bacterial blight resistance genes (*xa5*, *xa13* and *Xa21*) using marker-assisted selection into *indica* rice cultivar PR106. Theor. Appl. Genet..

[35] Sundaram R.M., Vishnupriya M.R., Biradar S.K., Laha G.S., Reddy G.A., Rani N.S., Sarma N.P., Sonti R.V. (2008). Marker assisted introgression of bacterial blight resistance in Samba Mahsuri, an elite indica rice variety. Euphytica.

[36] Ellur R.K., Khanna A., Yadav A., Pathania S., Rajashekara H., Singh V.K., Gopala Krishnan S., Bhowmick P.K., Nagarajan M., Vinod K.K. (2016). Improvement of Basmati rice varieties for resistance to blast and bacterial blight diseases using marker assisted backcross breeding. Plant Sci..

[37] Baliyan N., Malik R., Rani R., Mehta K., Vashisth U., Dhillon S., Boora K.S. (2018). Integrating marker-assisted background analysis with foreground selection for pyramiding bacterial blight resistance genes into Basmati rice. Comptes Rendus Biol..

[38] Rekha G., Abhilash Kumar V., Gokulan C.G., Koushik M.B.V.N., Laxmi Prasanna B., Kulkarni S., Aleena D., Harika G., Hajira S.K., Pranathi K. (2022). DRR Dhan 58, a seedling stage salinity tolerant NIL of improved Samba Mahsuri shows superior performance in multi-location trials. Rice.

[39] Shao Y., Peng Y., Mao B., Lv Q., Yuan D., Liu X., Zhao B. (2020). Allelic variations of the *Wx* locus in cultivated rice and their use in the development of hybrid rice in China. PLoS ONE.

[40] Neelam K., Thakur S., Neha Yadav I.S., Kumar K., Dhaliwal S.S., Singh K. (2017). Novel alleles of phosphorus-starvation tolerance 1 gene (*PSTOL1*) from *Oryza rufipogon* confers high phosphorus uptake efficiency. Front. Plant Sci..

[41] He S., Liang S., Meng L., Cao L., Ye G. (2023). Sparse phenotyping and haplotype-based models for genomic prediction in rice. Rice.

[42] Kumari A., Sharma D., Sharma P., Sahil, Wang C., Verma V., Patil A., Imran M., Singh M.P., Kumar K. (2023). Meta-QTL and haplo-pheno analysis reveal superior haplotype combinations associated with low grain chalkiness under high temperature in rice. Front. Plant Sci..

[43] Shen S., Xu S., Wang M., Ma T., Chen N., Wang J., Zheng H., Yang L., Zou D., Xin W. (2023). BSA-Seq for the identification of major genes for EPN in rice. Int. J. Mol. Sci..

[44] Liu Y., Xin W., Chen L., Liu Y., Wang X., Ma C., Zhai L., Feng Y., Gao J., Zhang W. (2024). Genome-wide association analysis of effective tillers in rice under different nitrogen gradients. Int. J. Mol. Sci..

[45] Jiao Y., Wang Y., Xue D., Wang J., Yan M., Liu G., Dong G., Zeng D., Lu Z., Zhu X. (2010). Regulation of *OsSPL14* by OsmiR156 defines ideal plant architecture in rice. Nat. Genet..

[46] Sasaki A., Ashikari M., Ueguchi-Tanaka M., Itoh H., Nishimura A., Swapan D., Ishiyama K., Saito T., Kobayashi M., Khush G.S. (2002). A mutant gibberellin-synthesis gene in rice. Nature.

[47] Xue W., Xing Y., Weng X., Zhao Y., Tang W., Wang L., Zhou H., Yu S., Xu C., Li X. (2008). Natural variation in *Ghd7* is an important regulator of heading date and yield potential in rice. Nat. Genet..

[48] Liu G., Qiu D., Lu Y., Wu Y., Han X., Jiao Y., Wang T., Yang J., You A., Chen J. (2023). Identification of superior haplotypes and haplotype combinations for grain size- and weight-related genes for breeding applications in rice (*Oryza sativa* L.). Genes.

[49] Zhang Q., Xie J., Wang X., Liu M., Zhu X., Yang T., Khan N.U., Sun C., Li J., Zhang Z. (2022). Natural variation of RGN1a regulates grain number per panicle in japonica rice. Front. Plant Sci..

[50] Liu Z., Sun H., Zhang Y., Du M., Xiang J., Li X., Chang Y., Sun J., Cheng X., Xiong M. (2023). Mining the candidate genes of rice panicle traits via a genome-wide association study. Front. Genet..

[51] Jaldhani V., Suman K., Rao D.S., Travis T., Rao I.S., Rathod S., Voleti S.R., Subrahmanyam D., Rao P.R., Barbadikar K.M. (2024). Superior haplotypes to enhance grain filling in rice (*Oryza sativa* L) identified through GWAS. Discov. Plants.

[52] Wang Y., Wang X., Zhai L., Zafar S., Shen C., Zhu S., Chen K., Wang Y., Xu J. (2024). A novel effective panicle number per plant 4 haplotype enhances grain yield by coordinating panicle number and grain number in rice. Crop J..

[53] Sun H., Yao Q., Hai M., Li T., Sun J., Liu Z., Ang Y., Zhao Y., Zhang Y., Cheng X. (2025). Elite alleles of EPE1 identified via genome-wide association studies increase panicle elongation length in rice. Rice.

[54] Li F., Kong D., Li Y., Li K., Xu J. (2026). Effects of haplotypes of the rice sucrose transporter genes *OsSWEET11* and *OsSWEET15* on grain traits in local Yunnan germplasm resources. Int. J. Mol. Sci..

[55] Singh N., Singh B., Rai V., Sidhu S., Singh A.K., Singh N.K. (2017). Evolutionary insights based on SNP haplotypes of red pericarp, grain size and starch synthase genes in wild and cultivated rice. Front. Plant Sci..

[56] Ashikari M., Sakakibara H., Lin S., Yamamoto T., Takashi T., Nishimura A., Angeles E.R., Qian Q., Kitano H., Matsuoka M. (2005). Cytokinin oxidase regulates rice grain production. Science.

[57] Huang X., Qian Q., Liu Z., Sun H., He S., Luo D., Xia G., Chu C., Li J., Fu X. (2009). Natural variation at the DEP1 locus enhances grain yield in rice. Nat. Genet..

[58] Fan C., Xing Y., Mao H., Lu T., Han B., Xu C., Li X., Zhang Q. (2006). GS3, a major QTL for grain length and weight and minor QTL for grain width and thickness in rice, encodes a putative transmembrane protein. Theor. Appl. Genet..

[59] Wang D., Wang J., Sun W., Qiu X., Yuan Z., Yu S. (2022). Verifying the breeding value of a rare haplotype of Chalk7, GS3, and Chalk5 to improve grain appearance quality in rice. Plants.

[60] Song X.-J., Huang W., Shi M., Zhu M.-Z., Lin H.-X. (2007). A QTL for rice grain width and weight encodes a previously unknown RING-type E3 ubiquitin ligase. Nat. Genet..

[61] Weng J., Gu S., Wan X., Gao H., Guo T., Su N., Lei C., Zhang X., Cheng Z., Guo X. (2008). Isolation and initial characterization of GW5, a major QTL associated with rice grain width and weight. Cell Res..

[62] Shomura A., Izawa T., Ebana K., Ebitani T., Kanegae H., Konishi S., Yano M. (2008). Deletion in a gene associated with grain size increased yields during rice domestication. Nat. Genet..

[63] Han Y., Hu Q., Gong N., Yan H., Khan N.U., Du Y., Sun H., Zhao Q., Peng W., Li Z. (2024). Natural variation in MORE GRAINS 1 regulates grain number and grain weight in rice. J. Integr. Plant Biol..

[64] Ma Z., Ming Y., Li J., Li H., Wang H., Zhu X., Xu Y., Zhao Y., Hu Q., Liu R. (2025). Natural variation in GNP3 determines grain number and grain yield in rice. Nat. Commun..

[65] Hu Q., Zhao Z., Ma L., Xia H., Ma Z., Xu P., Wang X., Zhu R., Zhao Y., Guo H. (2025). Natural variation of GNP2 enhances grain number to benefit rice yield. Nat. Commun..

[66] Zhao Y., Gao J., Wang X., Rashid M.A.R., Wu Z., Ma Z., Wu H., Xu B., Wu Z., Gu Y. (2025). Elite haplotype of STRONG1 enhances rice yield by improving lodging resistance, panicle and plant architecture. Nat. Commun..

[67] Yang B., Xu S., Xu L., You H., Xiang X. (2018). Effects of *Wx* and its interaction with SSIII-2 on rice eating and cooking qualities. Front. Plant Sci..

[68] Selvaraj R., Singh A.K., Singh V.K., Abbai R., Habde S.V., Singh U.M., Kumar A. (2021). Superior haplotypes towards development of low glycemic index rice with preferred grain and cooking quality. Sci. Rep..

[69] Liang C., Bu Y., Xu H., Ma X., Zhang X., Hu T., Xiang X., Hu Y., Xu L. (2026). QTL mapping for different resistant starch subtypes identified a superior haplotype balancing high RS content and relatively good eating and cooking qualities in rice. Front. Plant Sci..

[70] Zhang G.-M., Zheng T.-Q., Chen Z., Wang Y.-L., Wang Y., Shi Y.-M., Wang C.-C., Zhang L.-Y., Ma J.-T., Deng L.-W. (2018). Joint exploration of favorable haplotypes for mineral concentrations in milled grains of rice (*Oryza sativa* L.). Front. Plant Sci..

[71] Rohilla M., Mazumder A., Chowdhury D., Bhardwaj R., Kumar Mondal T. (2024). Understanding natural genetic variation for nutritional quality in grain and identification of superior haplotypes in deepwater rice genotypes of Assam, India. Gene.

[72] He L., Sui Y., Che Y., Liu L., Liu S., Wang X., Cao G. (2024). New insights into the genetic basis of lysine accumulation in rice revealed by multi-model GWAS. Int. J. Mol. Sci..

[73] Zhang L., Yu J., Deng B., Peng Y., Shao Y., Bao J. (2025). Genotypic variation and genetic control of phenolic compounds and antioxidant activity in Shanlan upland rice landrace. Int. J. Mol. Sci..

[74] Zhou Z., Yang Z., Zhang Q., Wang X., Chen Q., Yu X., Zhou Y., Ouyang Y., Zhang J., Xie W. (2026). Integrative multi-omics analysis reveals the domestication mechanism of black rice. Natl. Sci. Rev..

[75] Hu Z., Liu H., Guo M., Han X., Li Y., Chen R., Guo Y., Yang Y., Sun S., Zhou Y. (2025). Natural variation of an E3 ubiquitin ligase encoding gene Chalk9 regulates grain chalkiness in rice. Nat. Commun..

[76] Cao N., Zhou W., Zhao F., Jiao G., Xie L., Lu A., Wu J., Zhu M., Liu Y., Yu J. (2025). OsGATA7 and SMOS1 cooperatively determine rice taste quality by repressing OsGluA2 expression and protein biosynthesis. Nat. Commun..

[77] Peng B., Kong H., Li Y., Wang L., Zhong M., Sun L., Gao G., Zhang Q., Luo L., Wang G. (2014). OsAAP6 functions as an important regulator of grain protein content and nutritional quality in rice. Nat. Commun..

[78] Yang Y., Guo M., Sun S., Zou Y., Yin S., Liu Y., Tang S., Gu M., Yang Z., Yan C. (2019). Natural variation of OsGluA2 is involved in grain protein content regulation in rice. Nat. Commun..

[79] Singh P., Sundaram K.T., Vinukonda V.P., Venkateshwarlu C., Paul P.J., Pahi B., Gurjar A., Singh U.M., Kalia S., Kumar A. (2024). Superior haplotypes of key drought-responsive genes reveal opportunities for the development of climate-resilient rice varieties. Commun. Biol..

[80] Yuan J., Wang X., Zhao Y., Khan N.U., Zhao Z., Zhang Y., Wen X., Tang F., Wang F., Li Z. (2020). Genetic basis identification of candidate genes for salt tolerance in rice by GWAS. Sci. Rep..

[81] Min M.-H., Maung T.Z., Cao Y., Phitaktansakul R., Lee G.-S., Chu S.-H., Kim K.-W., Park Y.-J. (2021). Haplotype analysis of BADH1 by next-generation sequencing reveals association with salt tolerance in rice during domestication. Int. J. Mol. Sci..

[82] Herwibawa B., Lekklar C., Chadchawan S., Buaboocha T. (2024). Association of a specific OsCULLIN3c haplotype with salt stress responses in local Thai rice. Int. J. Mol. Sci..

[83] James M., Tyagi W., Magudeeswari P., Neeraja C.N., Rai M. (2024). Genome-wide association-based identification of alleles, genes and haplotypes influencing yield in rice (*Oryza sativa* L.) under low-phosphorus acidic lowland soils. Int. J. Mol. Sci..

[84] Miao S., Lu J., Zhang G., Jiang J., Li P., Qian Y., Wang W., Xu J., Zhang F., Zhao X. (2024). Candidate genes and favorable haplotypes associated with iron toxicity tolerance in rice. Int. J. Mol. Sci..

[85] Aung K.M., Oo W.H., Maung T.Z., Min M.-H., Somsri A., Nam J., Kim K.-W., Nawade B., Lee C.-Y., Chu S.-H. (2023). Genomic landscape of the OsTPP7 gene in its haplotype diversity and association with anaerobic germination tolerance in rice. Front. Plant Sci..

[86] Alam S., Sundaram K.T., Singh U.M., Srinivas Prasad M., Laha G.S., Sinha P., Singh V.K. (2024). Superior haplotypes towards the development of blast and bacterial blight-resistant rice. Front. Plant Sci..

[87] Sun X., Xiong H., Jiang C., Zhang D., Yang Z., Huang Y., Zhu W., Ma S., Duan J., Wang X. (2022). Natural variation of DROT1 confers drought adaptation in upland rice. Nat. Commun..

[88] Wang H., Ye T., Guo Z., Yao Y., Tu H., Wang P., Zhang Y., Wang Y., Li X., Li B. (2024). A double-stranded RNA binding protein enhances drought resistance via protein phase separation in rice. Nat. Commun..

[89] Yu J., Zhu C., Xuan W., An H., Tian Y., Wang B., Chi W., Chen G., Ge Y., Li J. (2023). Genome-wide association studies identify OsWRKY53 as a key regulator of salt tolerance in rice. Nat. Commun..

[90] Xu Y., Zhang L., Ou S., Wang R., Wang Y., Chu C., Yao S. (2020). Natural variations of SLG1 confer high-temperature tolerance in indica rice. Nat. Commun..

[91] Lin Y., Zhu Y., Cui Y., Qian H., Yuan Q., Chen R., Lin Y., Chen J., Zhou X., Shi C. (2023). Identification of natural allelic variation in TTL1 controlling thermotolerance and grain size by a rice super pan-genome. J. Integr. Plant Biol..

[92] Li J., Guo H., Lou Q., Zeng Y., Guo Z., Xu P., Gu Y., Gao S., Xu B., Han S. (2025). Natural variation of indels in the CTB3 promoter confers cold tolerance in japonica rice. Nat. Commun..

[93] Deng Y., Zhai K., Xie Z., Yang D., Zhu X., Liu J., Wang X., Qin P., Yang Y., Zhang G. (2017). Epigenetic regulation of antagonistic receptors confers rice blast resistance with yield balance. Science.

[94] Zhao Y., Huang J., Wang Z., Jing S., Wang Y., Ouyang Y., Cai B., Xin X.-F., Liu X., Zhang C. (2016). Allelic diversity in an NLR gene *BPH9* enables rice to combat planthopper variation. Proc. Natl. Acad. Sci. USA.

[95] Wang Q., Liu Y., He J., Zheng X., Hu J., Liu Y., Dai H., Zhang Y., Wang B., Wu W. (2014). STV11 encodes a sulphotransferase and confers durable resistance to rice stripe virus. Nat. Commun..

[96] Singh A., Singh Y., Mahato A.K., Jayaswal P.K., Singh S., Singh R., Yadav N., Singh A.K., Singh P.K., Singh R. (2020). Allelic sequence variation in the Sub1A, Sub1B and Sub1C genes among diverse rice cultivars and its association with submergence tolerance. Sci. Rep..

[97] Bhat J.A., Yu D., Bohra A., Ganie S.A., Varshney R.K. (2021). Features and applications of haplotypes in crop breeding. Commun. Biol..

[98] Thomson M.J., Singh N., Dwiyanti M.S., Wang D.R., Wright M.H., Perez F.A., DeClerck G., Chin J.H., Malitic-Layaoen G.A., Juanillas V.M. (2017). Large-scale deployment of a rice 6 K SNP array for genetics and breeding applications. Rice.

[99] Steele K., Quinton-Tulloch M., Vyas D., Witcombe J. (2025). Thousands of trait-specific KASP markers designed for diverse breeding applications in rice (*Oryza sativa*). G3 Genes Genomes Genet..

[100] Steele K.A., Quinton-Tulloch M.J., Amgai R.B., Dhakal R., Khatiwada S.P., Vyas D., Heine M., Witcombe J.R. (2018). Accelerating public sector rice breeding with high-density KASP markers derived from whole genome sequencing of *indica* rice. Mol. Breed..

[101] Sun C., Hu Z., Zheng T., Lu K., Zhao Y., Wang W., Shi J., Wang C., Lu J., Zhang D. (2017). RPAN: Rice pan-genome browser for ~3000 rice genomes. Nucleic Acids Res..

